# Distinct roles of RECQL5 in RAD51-mediated fork reversal and transcription elongation

**DOI:** 10.1093/nar/gkaf1019

**Published:** 2025-10-14

**Authors:** Tarun Nagraj, Satyaranjan Sahoo, Shariva Kadupatil, Ganesh Nagaraju

**Affiliations:** Department of Biochemistry, Indian Institute of Science, Bangalore 560012, India; Department of Biochemistry, Indian Institute of Science, Bangalore 560012, India; Department of Biochemistry, Indian Institute of Science, Bangalore 560012, India; Department of Biochemistry, Indian Institute of Science, Bangalore 560012, India

## Abstract

RECQL5 helicase has been implicated in the regulation of homologous recombination (HR), replication stress responses, transcription elongation, and resolution of transcription-replication conflicts. However, the underlying mechanism by which RECQL5 regulates multiple functions in genome maintenance is obscure. Here, we find that RECQL5 localizes to the stalled fork sites and restricts RAD51-mediated excessive fork reversal to promote unrestrained DNA synthesis. The replication defect in the absence of RECQL5 can be rescued by co-depletion of SMARCAL1/ZRANB3/HLTF/FBH1 fork remodelers and expression of HR-defective mutants of RAD51. The RAD51 regulation at the stalled fork sites by RECQL5 requires its binding to PCNA, RAD51, and helicase activity and is independent of its interaction with RNAPII. Notably, the RECQL5 mutant devoid of its interaction with RAD51 regulates transcription elongation comparable to that of wild-type RECQL5. Collectively, our data demonstrates that RECQL5 distinctly regulates transcription elongation and RAD51-mediated fork remodelling to safeguard the replicating genomes.

## Graphical Abstract

**Graphical Abstract:**
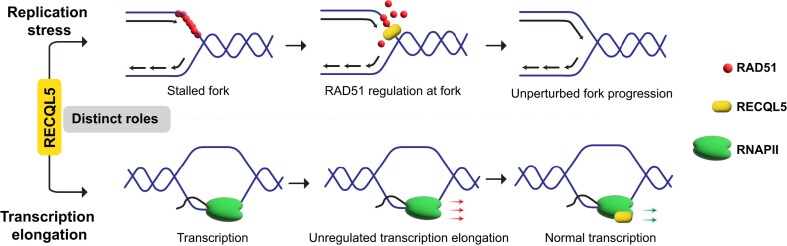

## Introduction

Error-free genome duplication is crucial for the maintenance of genome stability and tumour suppression. Nonetheless, DNA replication is challenged by various obstacles due to template damage, secondary structures, DNA-bound proteins, and nucleotide alterations [1, 2]. The stalled forks elicit a replication stress response, which facilitates remodelling of stalled forks via replication fork reversal (RFR) [3, 4]. RAD51 recombinase and SNF2 family translocases SMARCAL1, ZRANB3, and HLTF play an important role in remodelling the stalled forks [4, 5]. The reversed forks slow down DNA replication, stabilize the stalled forks from nucleolytic degradation, and promote the restart of stalled replication [6–9]. BRCA1, BRCA2, RAD51, and RAD51 paralogues participate in the repair of DNA double-strand breaks (DSBs) by homologous recombination (HR) [10–18]. In addition to their canonical role in HR-mediated DSB repair, these proteins participate in the stabilization of stalled forks from MRE11-, DNA2-, and EXO1-mediated degradation [19–26]. Although RFR is a protective mechanism to maintain genome stability, excessive fork reversal can be detrimental to cells, leading to genome instability [4, 27]. Indeed, fork reversal in the absence of BRCA2 and other fork-stabilizing factors has been shown to cause chromosomal aberrations, survival defects, and chemosensitivity of BRCA2-deficient cells [24, 28–30].

RecQ family helicases play crucial roles in multiple cellular functions, including DNA replication, transcription, and DNA repair, thereby maintaining genome stability and suppressing tumorigenesis [31–35]. Human RECQL5 belongs to the RecQ family of DNA helicases [32, 35]. RECQL5 localizes to the sites of DSBs, and mice cells lacking Recql5 exhibit a high rate of sister chromatid exchanges (SCEs), suggesting an HR regulation by RECQL5 during DSB repair [36]. Consistently, RECQL5-deficient cells display elevated frequencies of HR, and purified RECQL5 interacts with RAD51 and disrupts RAD51 filaments from single-stranded DNA (ssDNA) [37, 38]. In addition to its role in HR-mediated DSB repair, RECQL5 also participates in replication stress responses, transcription elongation, and preventing transcription-replication collisions (TRCs). RECQL5-deficient cells exhibit hypersensitivity to camptothecin (CPT) and a significant reduction in DNA replication in response to CPT-induced DNA damage [39]. RECQL5 interacts with RNAPII and inhibits transcription elongation in a manner dependent on its ATPase/helicase activity [40–42]. A further study showed that RECQL5 prevents genome instability associated with transcription stress in human cells. RECQL5 loss resulted in elevated transcription elongation rates, which are accompanied by pausing, arrest, and backtracking of RNAPII, leading to transcription stress [43]. Notably, chromosomal breakpoints at transcribed regions of genes and common fragile sites (CFSs) were found in RECQL5-deficient cells [43]. The RNAPII pausing also generates replication stress by replisome stalling in actively transcribed genes, resulting in TRCs [44–46]. Recent studies show the participation of RECQL5 in the resolution of TRCs. RECQL5 prevents replication fork stalling at RNAPI and RNAPII transcribed genes [47]. RECQL5 promotes MUS81-dependent mitotic DNA synthesis (MiDAS) at CFSs upon Ser727 phosphorylation by CDK1 [48]. RECQL5 facilitates the restart of stalled forks at R-loop sites in a manner dependent on MUS81-mediated fork cleavage and religation by LIG4/XRCC4 [49]. However, the underlying mechanism by which RECQL5 participates in replication stress responses is not completely understood.

Here, we find that RECQL5 prevents RAD51-mediated excessive fork remodelling to promote genome duplication. RECQL5’s association with PCNA, its helicase activity, and its interaction with RAD51 are required for suppressing excessive fork reversal by RAD51. Co-depletion of SMARCAL1/ZRANB3/HLTF/FBH1 fork remodelers and expression of HR-defective mutants of RAD51 rescues replication defects in RECQL5-deficient cells. Interestingly, the interaction of RECQL5 with RNAPII is dispensable for RAD51 regulation at the stalled fork sites. Notably, the RECQL5 mutant, defective in its binding to RAD51, was competent for regulating transcription elongation. These data unravel the distinct roles of RECQL5 in regulating RAD51-mediated excessive fork reversal and transcriptional elongation to facilitate genome duplication and maintain genome integrity.

## Materials and methods

### Cell lines and cell culture

Human cell lines U2OS, U2OS-SCR24, and hTERT-RPE1 were grown in Dulbecco’s modified Eagle’s medium with high glucose supplemented with 10% FBS (fetal bovine serum) (Gibco), 1% GlutaMAX (Gibco), and penicillin–streptomycin (Sigma–Aldrich) at 37°C in a humidified air incubator containing 5% CO_2_. All experiments were performed between 3rd and 15th passages. U2OS-SCR24 cells were propagated under puromycin selection (2 μg/ml; Sigma–Aldrich). U2OS-SCR24 cells were a kind gift from Prof. Ralph Scully, and hTERT-RPE1 cells were a kind gift from Dr Sachin Kotak.

### DNA constructs and transfections

All the short hairpin RNAs (shRNAs) were from previously reported siRNA sequences (Supplementary Table S1) and were cloned into the pRS shRNA vector and confirmed by sequencing. The plasmid encoding full-length wild-type (WT) human RECQL5 was a kind gift from Prof. Vilhelm Bohr and was subcloned into pcDNA3β expression vector by polymerase chain reaction amplification with a C-terminal FLAG tag. The shRNA-resistant WT RECQL5 was generated by introducing silent mutations at the RECQL5 shRNA#1 target sequence. All the mutants were generated by site-directed mutagenesis and cloned into the pcDNA3β vector. The sequence of the primers used for mutagenesis is mentioned in Supplementary Table S2. XRCC3, XRCC2, and RAD51 mutant constructs used were previously reported [50]. The design and construction of the sister chromatid recombination (SCR) reporter and I-SceI expression vector have been described previously [51]. All plasmid transfections for transient depletion/expression were performed using a Bio-Rad Gene Pulsar Xcell (260 V, 1050 μF). Cells were recovered in fresh media 6–8 h post-transfection. Cells were processed for the indicated treatments/experiments 24–30 h after transfection.

### Western blotting

Cells were harvested by trypsinization and lysed in RIPA buffer [150 mM NaCl, 1% Igepal CA-630, 0.5% sodium deoxycholate, 0.1% SDS, 50 mM Tris–HCl (pH 8.0)] supplemented with protease inhibitor (cOmplete Protease Inhibitor Cocktail, Roche). Twenty-five to eighty micrograms of proteins were resolved on 6%–10% sodium dodecyl sulfate–polyacrylamide gel electrophoresis (SDS–PAGE) and transferred onto a PVDF membrane (Millipore) using a Bio-Rad transfer apparatus (Bio-Rad Trans-Blot SD). The membranes were blocked using blocking buffer [5% skimmed milk (w/v) in PBST (0.1% Tween-20 in 1× PBS)] for 1 h at room temperature (RT). The membranes were incubated with indicated primary antibodies overnight (O/N) in blocking buffer at 4°C. After three washes with PBST, membranes were incubated with HRP-conjugated secondary antibodies (1:10 000; SCBT) for 1 h at RT. Later, membranes were washed in PBST, developed with chemiluminescent HRP substrate (Millipore), and imaged using a Chemidoc (Bio-RAD Chemidoc Imaging System). The details of primary antibodies used for immunoblotting in this study are rabbit anti-RECQL5 (1:1000, Abcam), mouse anti-MCM3 (1:500, SCBT), mouse anti-HLTF (1:250, SCBT), mouse anti-LAMIN (1:1000, SCBT), rabbit anti-ZRANB3 (1:500, Abcam), rabbit anti-SMARCAL1 (1:500, Abcam), mouse anti-FBH1 (1:200, SCBT), mouse anti-RAD51 (1:250, SCBT), mouse anti-RAD51C (1:250, SCBT), mouse anti-XRCC2 (1:250, SCBT), mouse anti-XRCC3 (1:250, SCBT), mouse anti-FLAG (1:2000, Sigma), mouse anti-HA (1:2000, Roche), rabbit anti-RTEL1 (1:500, Invitrogen), mouse anti-β ACTIN (1:1000, SCBT), mouse anti-α TUBULIN (1:1000, SCBT) and mouse anti-GAPDH (1:1000, SCBT).

### Immunofluorescence

Immunofluorescence was performed as described previously [52]. Exponentially growing cells were seeded onto coverslips after indicated transfections and allowed to settle O/N. After the indicated treatments, cells were washed twice with PBS and pre-extracted with 0.5% Triton X-100 in 1× PBS for 5 min on ice, followed by fixation with 3.7% formaldehyde in 1× PBS for 12 min at RT. For native BrdU staining, cells were incubated with 25 μM BrdU for 24 h; later, cells were washed thrice with 1× PBS and then treated with HU. After three 1× PBS washes, coverslips were incubated with a blocking buffer [3% bovine serum albumin (BSA) in PBST] for 1 h. Later, cells were incubated with primary antibodies diluted in a blocking buffer for 2 h at RT. After two washes with blocking buffer, cells were incubated with respective FITC/TRITC-conjugated secondary antibodies for 1 h at RT. Cells were washed twice with PBST and stained with DAPI (1 μg/ml; Sigma) for 5 min before mounting onto slides with Mowiol 4–88 (Sigma). Images were acquired using an apotome microscope (Zeiss Axio Observer) at 63× magnification and processed and quantified using FIJI software. Primary antibodies used for immunofluorescence: mouse anti-H2AX (pS139) (1:2000, BD Biosciences), rabbit anti-53BP1 (1:1000, Novus Biologicals), rabbit anti-RAD51 (1:500, Abcam), rat anti-BrdU (1:250, Abcam), goat anti-mouse FITC (1:1000, Sigma), and goat anti-rabbit (1:500, Sigma).

### SIRF assay

SIRF was performed as described previously with minor changes [53, 54]. Briefly, 6–8 h after indicated transfection, cells were seeded onto coverslips and allowed to settle for at least 12 h. After indicated EdU labelling, HU treatment, and recovery conditions, cells were washed thrice with 1× PBS. Cytoplasmic and nucleoplasmic proteins were washed to enrich chromatin-bound proteins by incubating cells with pre-extraction buffer (ice-cold 0.5% Triton X-100 in 1× PBS) for 5 min on ice and fixed in 3.7% formaldehyde in 1× PBS for 12 min at RT. Later, cells were washed thrice with 1× PBS and permeabilized with 0.5% Triton X-100 in PBS for 10 min at RT. After one 1× PBS wash, coverslips were incubated with a freshly prepared Click reaction cocktail (2 mM CuSO4, 10 μM biotin-azide, and 100 mM sodium ascorbate in PBS) for 1 h in a humidified chamber at RT. Coverslips were incubated in blocking buffer (10% goat serum and 0.1% Triton X-100 in PBS) for 1 h at RT, followed by incubation with primary antibodies at 4°C diluted in blocking buffer, O/N along with appropriate anti-biotin antibody. To detect RAD51, RAD51 paralogue RAD51C, and XRCC3, primary antibody incubation was done at 37°C for an additional 1 h after O/N incubation. PLA was performed according to the manufacturer’s recommendations (Duolink Proximity Ligation assay kit, Merck). Briefly, after three washes with 1× PBS, coverslips were incubated with anti-mouse minus and anti-rabbit plus probes in a humidified chamber for 1 h at 37°C. The cells were washed twice with PLA wash buffer A [10 mM Tris–HCl (pH 7.4), 150 mM NaCl, 0.05% Tween-20] followed by ligation in a humidified chamber for 30 min at 37°C and then washed with PLA wash buffer A and incubated with amplification buffer with polymerase at 37°C for 100 min at 37°C. Cells were washed twice with PLA wash buffer B [200 mM Tris–HCl (pH 7.4), 100 mM NaCl] and then stained with DAPI (1 μg/ml; Sigma–Aldrich) for 5 min before mounting onto slides with Mowiol 4–88 (Sigma). Images were acquired using an Apotome microscope (Zeiss Axio Observer) at 63× magnification, and SIRF foci were quantified using FIJI software. Primary antibodies used for SIRF assay: mouse anti-biotin (1:500, Invitrogen), rabbit anti-biotin (1:1000, CST), rabbit anti-RAD51 (1:500, Abcam), rabbit anti-RAD51C (1:500, Abcam), mouse anti-XRCC3 (1:100, SCBT), rabbit anti-RTEL1 (1:75, Invitrogen), rabbit anti-RECQL5 (Abcam, 1:500), and rabbit anti-FLAG (1:500, CST).

### DNA fibre spreads

Cells were pulse-labelled with 50 μM CldU (Sigma) and 250 μM IdU (Sigma) as indicated in the sketches. Replication was halted by adding chilled 1× PBS and incubating the plates on ice for 5 min. Cells were later harvested, counted, and resuspended in chilled PBS at 1 × 10^6^ cells/ml. Labelled and unlabelled cells were mixed in a 2:1 ratio, and 3 μl of the cell mixture was mixed with 7 μl DNA lysis buffer [200 mM Tris–HCl (pH 7.4), 50 mM ethylenediamine tetraacetic acid (EDTA) (pH 8.0), 0.5% SDS] and allowed to stand for 7 min. Fibres were stretched by inclining slides to 30°–45°, air dried for ∼20 min, fixed in methanol:acetic acid (3:1) solution, and incubated at 4°C O/N. The following day, DNA was denatured by incubating in 2.5 N HCl for 80 min. After serial washes in 1× PBS, slides were incubated in a blocking buffer (2% BSA, 0.1% Tween-20 in PBS) for 1 h. Later, slides were incubated in primary antibodies for 2.5 h diluted in a blocking buffer. Slides were washed thrice in PBST (0.2% Tween-20 in PBS) and incubated again in a blocking buffer for 15 min. Slides were then incubated in secondary antibodies for 1 h, washed thrice with PBST, once with 1× PBS, and air-dried for 30 min in the dark and later mounted using Mowiol 4–88 (Sigma). DNA fibres were visualized using an Apotome microscope (Zeiss Axio Observer) at 40× or 63× magnification. DNA fibre length was measured using FIJI software. Replication rate was calculated by (2.59 kb/μm) X fibre length in μm/min. Antibodies used for DNA fibre assay: rat anti-BrdU (1:500, Abcam), mouse anti-BrdU (1:250, BD Biosciences), rabbit anti-mouse IgG Alexa Fluor 488 (1:500, Abcam), and donkey anti-rat IgG Alexa Fluor 594 (1:500, Abcam).

### Transcription elongation assay

The transcription elongation assay was performed as described previously [55]. Briefly, cells were seeded onto glass coverslips 6–8 h after the indicated transfections. Thirty hours post-transfection, RNAPI inhibition was carried out by incubating cells with 0.05 μg/ml actinomycin D (Sigma) for 3 h. Cells were pulsed with 1 mM EU (Sigma) containing inhibitor at 1.5 h before the endpoint. After two 1× PBS washes, cells were fixed with 4% formaldehyde in PBS for 10 min and permeabilized with 0.5% Triton X-100 for 15 min at RT. After one 1× PBS wash, coverslips were incubated with a freshly prepared Click reaction cocktail [100 mM Tris–HCl (pH 8.5), 5 mM CuSO4, 10 μM Alexa Fluor 488 azide, and 100 mM sodium ascorbate] for 1 h in a humidified chamber at RT. Cells were then washed once with 100 mM Tris (pH 7.5) and stained with DAPI (1 μg/ml) for 5 min and mounted with Mowiol 4–88 (Sigma). Images were acquired using an apotome microscope (Zeiss Axio Observer) at 20× magnification. Mean nuclear fluorescence intensity was quantified after subtracting background signals using FIJI software.

### HR assay

HR assay was performed as described previously [56]. Briefly, U2OS-SCR24 cells were transfected with indicated constructs. After 24 h, cells were transfected with 25 μg of I-SceI plasmid/10^6^ cells along with the indicated constructs. Cells were harvested after 72 h, and GFP+ cells were analysed using FACS. The percentage of GFP+ cells was normalized with transfection efficiency (∼75–80%) for each experiment. The spontaneous GFP+ percentage (0.02%) was subtracted from absolute GFP frequency to obtain I-SceI-induced HR frequencies. To estimate spontaneous HR events, the percentage of GFP+ cells was analysed as mentioned earlier without I-SceI transfection. For HU-induced HR frequency, cells were transfected with the indicated plasmid constructs. Twenty-four hours post-transfection, cells were treated with 100 μM HU for 24 h. Cells were treated with 50 μM mirin 4 h before the indicated HU treatment. Cells were harvested, and GFP+ cells were scored by FACS using a CytoFLEX flow cytometer (Beckman Coulter). At least 10 000 cells were analysed for each biological repeat. The percentage of GFP+ cells was normalized with transfection efficiency.

### Cell survival

Cells (5000/well) were plated in 24-well plates 8 h post-transfection and allowed to settle for at least 12 h. Thirty hours post-transfection, cells were treated with indicated drugs and allowed to grow for 7 days. Later, cell survival was monitored by MTT assay (0.1 mg/ml: Sigma) using a microplate reader (VersaMaxROM version 3.13). Percent cell survival was calculated as (treated cells/untreated cells) × 100.

### Neutral comet assay

Silane-Prep slides (Sigma; S4651) were coated with 1% agarose in Milli-Q and air-dried at least 24 h before the experiment. After indicated treatments, cells were harvested in 1× PBS. About 50,000 cells were resuspended in 100 μL of 1% LMP agarose (Sigma) in 1× PBS and spread onto pre-coated slides. Slides were lysed in chilled lysis buffer [2.5 M NaCl, 0.1 M EDTA (pH 8.0), 100 mM Tris–HCl (pH 10.0), 1% N-lauryl sarcosine, 0.5% Triton X-100, 10% DMSO (dimethyl sulfoxide)] for O/N at 4°C. The next day, the slides were then washed thrice in chilled electrophoresis buffer [300 mM sodium acetate, 100 mM Tris–HCl (pH 8.0)] and were transferred to an electrophoresis tank filled with chilled electrophoresis buffer. The electrophoresis was performed at 0.5 V/cm for 1 h at 4°C. After washing slides twice in 1× PBS, they were fixed in absolute ethanol for 30 min at RT. Slides were then air-dried and stained with PI (2 μg/ml in 1× PBS, Sigma). Images were captured at 10× magnification, and the comet tail moment was measured using the OpenComet plugin in FIJI software.

### Metaphase spreads

After indicated transfections, cells were treated with 2 mM HU for 4 h and allowed to recover in fresh media for 24 h. Cells were incubated with 0.25 μg/mL Colcemid (KaryoMAX, Gibco) for the last 4 h of recovery to enrich for metaphase population. Cells were harvested, washed twice with 1× PBS, resuspended in pre-warmed hypotonic solution (75 mM KCl in MilliQ), and incubated in a 37°C water bath for 15 min. Later, cells were pelleted down at 160 × *g* for 10 min at 4°C and fixed with 5 ml of freshly prepared Carnoy’s fixative (3:1 ratio of methanol:acetic acid). Cell pellets were washed thrice with Canoy’s fixative, and the final pellet was resuspended in 100 μl of Canoy’s fixative. Fixed cells were lysed by dropping 100 μl of the cell suspension onto pre-chilled frosted slides, then incubating on a steaming water beaker for 2 min. Slides were air-dried and stained with Giemsa (1:20; Sigma) for 30 min at RT. Excess stain was washed off and air-dried O/N. Images were captured using an Olympus BX53 microscope at 100× magnification.

### *In situ* proximity ligation assay

Cells were seeded, pre-extracted and fixed as indicated in SIRF assays. After incubating coverslips in blocking buffer for 1 h at 37°C in a humidified chamber, primary antibody incubation was done O/N at 4°C. The following day, PLA was performed as per the manufacturer’s guidelines (NaveniFlex Cell Red, Navinci). Finally, nuclei were counter-stained with DAPI (1 μg/ml; Sigma–Aldrich) for 5 min before mounting onto slides with Mowiol 4–88 (Sigma). Images were acquired using an Apotome microscope (Zeiss Axio Observer) at 63× magnification, and PLA foci were quantified using FIJI software. Primary antibodies used for PLA reactions: anti-mouse PCNA (1:750; SCBT) and anti-rabbit RNAPIIS2P (1:5000, Abcam).

### Co-immunoprecipitation

Co-immunoprecipitation (Co-IP) experiments were performed as described previously but without benzonase treatment [52]. Briefly, cells were harvested upon indicated treatments and lysed with HEPES-Triton buffer [35 mM HEPES KOH (pH 7.6), 200 mM NaCl, 1 mM EDTA (pH 8.0), 0.75% Triton X-100, 8% glycerol, supplemented with protease inhibitor cocktail (Sigma)] on ice for 20 min. One milligram of clarified lysates was incubated with 3 μg of antibody O/N on a rotating wheel at 4°C. The following day, the Co-IP reaction was incubated with 50 μl of BSA-blocked protein A beads for 4 h. Then, the IP was washed once with lysis buffer and once with IP wash buffer [100 mM Tris–HCl (pH 8), 300 mM NaCl, 1 mM EDTA (pH 8.0), 1% Triton X-100, 8% glycerol, supplemented with protease inhibitor cocktail (Sigma)]. Proteins were eluted by boiling the beads in 2× Laemmli for 15 min and proceeded for western blotting.

### Quantification and statistical analysis

All experiments were independently performed at least three times unless mentioned otherwise in the figure legends, and statistical parameters are mentioned in the figure legends. Data represent mean ± SD/SEM from at least three independent experiments. For IF and SIRF experiments, >80 cells were quantified for the experimental sample of each biological replicate. At least 100 fibres were quantified for each experimental sample of each biological replicate. The statistical analysis of all experiments was performed using GraphPad Prism V9.

## Results

### RECQL5 helicase promotes genome-wide replication

**Figure 1.**
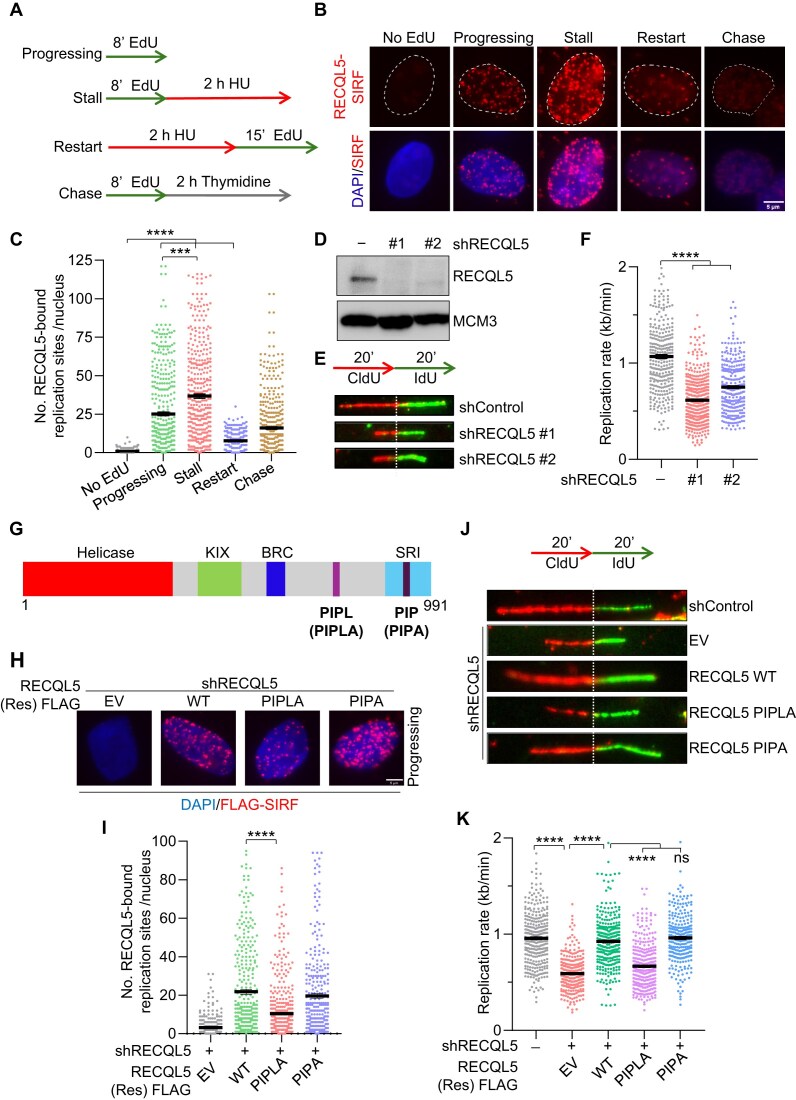
RECQL5’s association with the active replication forks facilitates genome-wide replication in human cells. (**A**) A schematic of the conditions analysed in the SIRF assay. No EdU serves as a negative control. (**B**) Representative images of chromatin-bound RECQL5-SIRF signals in U2OS cells in the conditions described in panel (A). Scale bar, 5 μm. (**C**) Quantification of chromatin-bound RECQL5-SIRF signals as shown in panel (B). A total of ≥300 nuclei were analysed for no EdU, progressing, stall, restart, and chase conditions across three biological replicates. Data are represented as mean ± SEM. Mann–Whitney *t*-test, *P< .05; **P< .001; ***P< .0001; ****P< .0001; ns, non-significant. (**D**) Representative immunoblot showing knockdown of RECQL5 in U2OS cells using two independent gene-specific shRNAs, #1 and #2. MCM3 serves as loading control. (**E**) (Top) Schematic of labelling scheme used for DNA fibre assay. (Bottom) Representative DNA fibre images to show fork slowdown in control and RECQL5-depleted U2OS cells. (**F**) Quantification of IdU track lengths as replication rate (kb/min) in control and RECQL5-depleted cells as shown in panel (E). A total of ≥300 DNA fibres were analysed across three biological replicates. Data are represented as mean ± SEM. Mann–Whitney *t*-test, *P< .05; **P< .001; ***P< .0001; ****P< .0001; ns, non-significant. (**G**) Domain architecture of RECQL5 helicase. Sites of mutations in PIP and PIPL motifs are indicated. (**H**) Representative images of chromatin-bound RECQL5-FLAG SIRF signals in RECQL5-depleted U2OS cells transfected with empty vector, shRNA#1 resistant WT, PIPL, and PIP motif mutants of RECQL5. Scale bar, 5 μm. (**I**) Quantification of chromatin-bound RECQL5-FLAG-SIRF signals at actively replicating sites as shown in panel (H). A total of ≥300 nuclei were analysed across three biological replicates. Data are represented as mean ± SEM. Mann–Whitney *t*-test, *P< .05; **P< .001; ***P< .0001; ****P< .0001; ns, non-significant. (**J**) (Top) Schematic of labelling scheme used for DNA fibre assay. (Bottom) Representative DNA fibre images are used to show the replication rate in control and RECQL5-depleted U2OS cells transfected with an empty vector and shRNA#1-resistant WT, PIPL, and PIP motif mutants of RECQL5. (**K**) Quantification of IdU track lengths as replication rate (kb/min) as shown in panel (J). A total of ≥300 DNA fibres were analysed across three biological replicates. Data are represented as mean ± SEM. Mann–Whitney *t*-test, *P< .05; **P< .001; ***P< .0001; ****P< .0001; ns, non-significant. Unless otherwise specified, shRNA1 was used to knock down RECQL5.

Besides regulating HR during DSB repair, RECQL5 also has been implicated in replication stress responses and suppressing TRCs [33, 34]. However, its precise function in regulating replication remains unclear. To examine whether RECQL5 localizes to actively replicating sites, we performed an *in situ* analysis of protein interactions at DNA replication forks (SIRF) [53], a PLA-based approach to effectively quantify protein interactions at nascent replication forks at a single-cell resolution. U2OS cells were pulse labelled with 5′-Ethynyl-2′-deoxyuridine (EdU) to mark the sites of nascent DNA. Further, cells were treated with hydroxyurea (HU), followed by recovery in EdU-containing media to study the fork stalling and restart dynamics, respectively. EdU pulse followed by thymidine chase was also included (Fig. 1A). We find that RECQL5 is associated with progressing, stalled, and restarted forks, with the stalled forks showing the highest enrichment (Fig. 1B and C). RECQL5-SIRF signals were substantially decreased upon depletion of RECQL5, establishing that the SIRF signals are specific (Fig. 1D and Supplementary Fig. S1A). Our observation aligns with the previous iPOND-SILAC quantitative mass spectrometry study that reported RECQL5 to be associated with active replisomes [57]. This observation indicates that RECQL5 is dynamically associated with active replication forks and is further enriched upon replication stalling.

As RECQL5 is associated with active forks, we performed DNA fibre analysis to determine the effect of RECQL5 depletion on DNA replication kinetics. We sequentially pulse labelled cells with 5-chloro-2′-deoxyuridine (CldU) and 5-iodo-2′-deoxyuridine (IdU) and measured the DNA tract lengths. RECQL5 depletion by two independent gene-specific shRNAs led to a marked decrease in the replication rate with a concomitant increase in fork asymmetry (Fig. 1D–F and Supplementary Fig. S1C). We confirmed that the phenotype is not cell type-specific, as silencing RECQL5 in hTERT-RPE1 cells also significantly decreased the replication rate (Supplementary Fig. S1B). The reduced replication rate in RECQL5-depleted cells could be partly due to frequent fork pausing and stalling. Since RECQL5 is also enriched at stalled and restarted forks (Fig. 1C), we then performed a fork restart assay to investigate the role of RECQL5 at the stalled forks. Control and RECQL5-depleted cells were labelled with CldU, followed by exposure with HU to induce fork stalling and recovery in fresh media containing IdU to label restarted forks. Indeed, RECQL5-depleted cells showed an increased frequency of stalled forks, suggesting a defect in fork restart (Supplementary Fig. S1D). Moreover, analysis of the restarted forks reveals that fork progression is considerably slower in RECQL5-depleted cells upon recovery from replication stress (Supplementary Fig. S1E). Defects in normal replication progression and fork protection mechanisms can lead to fork collapse and persistent DNA damage. The repair kinetics, as measured by clearance of γH2AX and 53BP1 foci upon treatment with HU, were significantly slower in the absence of RECQL5 (Supplementary Fig. S2A–C). Consistently, the tail moment from the neutral comet assay showed elevated DSBs in unchallenged and HU-treated RECQL5-depleted cells (Supplementary Fig. S2D). Moreover, RECQL5-depleted cells also exhibited a significant reduction in survival in response to HU and aphidicolin-induced replication stress (Supplementary Fig. S2E and F). These results suggest that RECQL5 promotes genome-wide DNA replication and maintains genome stability in both normal and replication stress conditions.

An *in vitro* study identified that RECQL5 interacts with PCNA through the PIP (PCNA interacting protein) motif located between 964 and 971 residues that overlap with the Set2–RPB1 interaction (SRI) domain [58] (Fig. 1G). RECQL5 also harbours a non-canonical PIPL (PIP-like) motif located between 761 and 768 residues [59] (Fig. 1G). However, a recent study showed that RECQL5 interacts with PCNA primarily through the PIPL motif [59]. To investigate whether the PIP or PIPL motif is critical for RECQL5-PCNA interaction at replisomes, we generated PIPA (Q964A, I967A, F970A, and F971A) and PIPLA (I764A, F767A, and F768A) (Fig. 1G) mutants and measured their association with active forks. We transiently expressed shRNA-resistant WT, PIPA, and PIPLA FLAG-tagged mutants (Supplementary Fig. S2G) in RECQL5-depleted U2OS cells and examined their association with progressing forks by scoring RECQL5-FLAG-SIRF foci. Our analysis revealed that the PIPA mutant was able to associate with the progressing fork at a comparable level to that of WT RECQL5 (Fig. 1H and I). In contrast, the PIPLA mutant showed a significant reduction in its association with the progressing fork compared to WT (Fig. 1H and I). Consistently, the expression of WT and PIPA RECQL5 mutants, but not the PIPLA mutant, rescued the replication rate in RECQL5-deficient cells (Fig. 1J and K). These observations suggest that RECQL5 promotes unrestrained DNA synthesis by interacting with PCNA via a non-canonical PIPL motif.

### RAD51-mediated unscheduled fork reversal leads to replication defects in the absence of RECQL5

**Figure 2.**
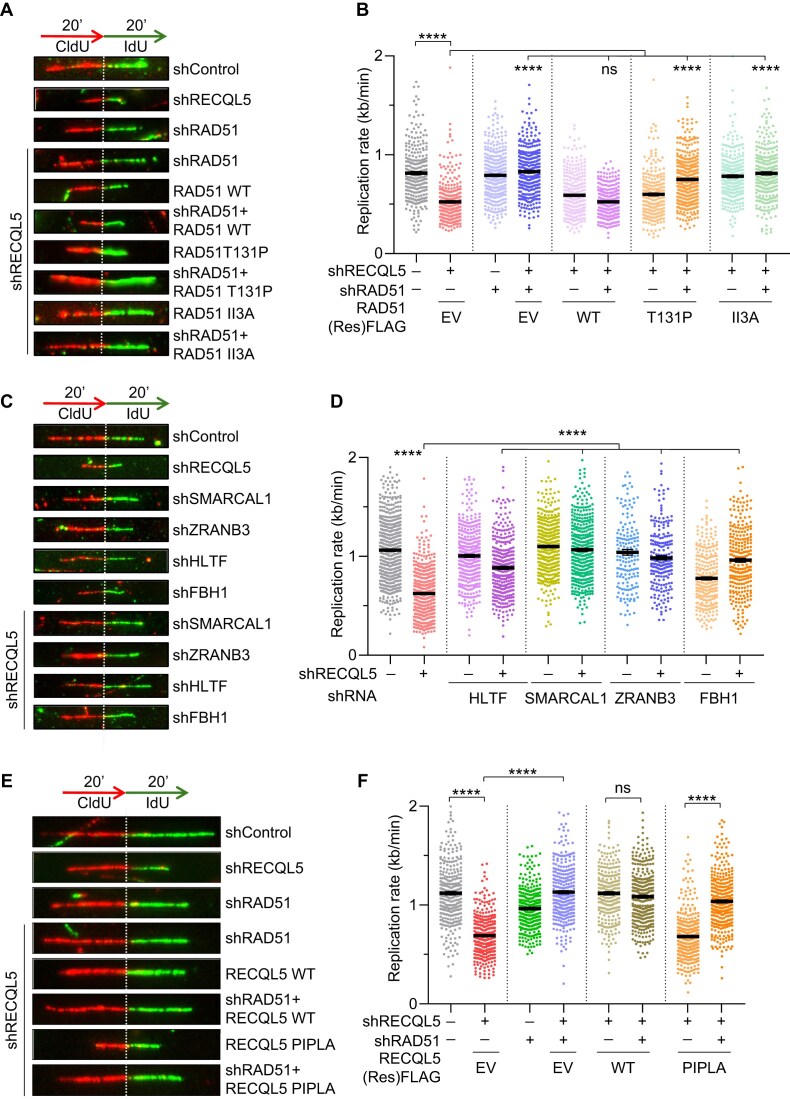
RAD51-mediated excessive fork remodelling leads to replication defects in RECQL5-depleted cells. (**A**) (Top) Schematic of labelling scheme used for DNA fibre assay. (Bottom) Representative DNA fibre images showing replication rate in indicated U2OS cells. (**B**) Quantification of IdU track lengths as replication rate (kb/min) for indicated conditions shown in panel (A). A total of ≥300 DNA fibres were analysed across three biological replicates. Data are represented as mean ± SEM. Mann–Whitney *t*-test, *P< .05; **P< .001; ***P< .0001; ****P< .0001; ns, non-significant. (**C**) (Top) Schematic of labelling scheme used for DNA fibre assay. (Bottom) Representative DNA fibre images to show replication rates in control, RECQL5 alone, and RECQL5 co-depleted with indicated fork reversal factors in U2OS cells. (**D**) Quantification of IdU track lengths as replication rate (kb/min) for indicated conditions shown in panel (C). A total of ≥300 DNA fibres were analysed across three biological replicates. Data are represented as mean ± SEM. Mann–Whitney *t*-test, *P< .05; **P< .001; ***P< .0001; ****P< .0001; ns, non-significant. (**E**) (Top) Schematic of labelling scheme used for DNA fibre assay. (Bottom) Representative DNA fibre images showing replication rate in indicated U2OS cells. (**F**) Quantification of IdU track lengths as replication rate (kb/min) for indicated conditions shown in panel (E). A total of ≥300 DNA fibres were analysed across three biological replicates. Data are represented as mean ± SEM. Mann–Whitney *t*-test, *P< .05; **P< .001; ***P< .0001; ****P< .0001; ns, non-significant.

RAD51 localizes to stalled forks and promotes fork reversal [5]. However, excessive or unregulated fork reversal can affect normal replication [50]. RECQL5 has been shown to disrupt RAD51 filaments from ssDNA [37]. The replication defect in RECQL5-depleted cells could be due to unregulated RAD51-mediated fork remodelling at the active/stalled forks. In such a scenario, co-depleting RAD51 in RECQL5-depleted cells should be able to rescue fork progression defects caused by RECQL5 deficiency. To test this, we co-depleted RAD51 in RECQL5 depletion background and analysed replication rates by DNA fibre assay. Indeed, silencing RAD51 in RECQL5-depleted cells (Supplementary Fig. S3A) rescued the decreased replication rate observed in RECQL5-deficient cells (Fig. 2A and B). RAD51 promotes fork reversal through its strand exchange activity [60]. We hypothesized that the expression of the strand exchange activity deficient RAD51 T131P and II3A mutants could restore the observed replication defects in RECQL5-deficient cells [61, 62]. Indeed, the expression of T131P RAD51 mutant rescued replication defects in RECQL5–RAD51 co-depleted cells (Fig. 2A, 2B, and Supplementary Fig. S3B). We also observed that the expression of RAD51 II3A mutant in RECQL5 alone and RECQL5–RAD51 co-depleted cells rescued the replication rate in the absence of RECQL5 (Fig. 2A, 2B, and Supplementary Fig. S3B).

RAD51 facilitates fork reversal in coordination with fork remodelling enzymes such as HLTF/ZRANB3/SMARCAL1/FBH1 [5, 63–65]. Co-depletion of HLTF/ZRANB3/SMARCAL1/FBH1 (Supplementary Fig. S3C–F) significantly restored the reduced replication rates observed in RECQL5-deficient U2OS and hTERT-RPE1 cells (Fig. 2C, 2D, and Supplementary Fig. S3G). Interestingly, silencing of ZRANB3/HLTF also reduced the frequency of DSBs observed in RECQL5-depleted cells (Supplementary Fig. S3H), suggesting that breaks were generated from uncontrolled fork reversal. Consistent with our observations, electron microscopy analysis from a recent study also showed a 2–3-fold increase in the reversed replication forks in the absence of replication stress in RECQL5-depleted U2OS cells [49]. As RECQL5 is recruited to active replication forks through its PIPL motif (Fig. 1H and I), we reasoned that loss of its interaction with PCNA in the RECQL5-PIPL mutant might promote excessive RAD51-mediated fork reversal, leading to replication defects. Indeed, cells expressing RECQL5-PIPL mutant exhibited a replication defect (Fig. 1J and K), which was rescued by co-depletion of RAD51 (Fig. 2E and F). Together, these data suggest that RECQL5 prevents RAD51-mediated fork reversal in unperturbed conditions to promote genome duplication.

### RECQL5 helicase regulates HR factors at the replication sites and prevents hyper-recombination to facilitate replication progression

**Figure 3.**
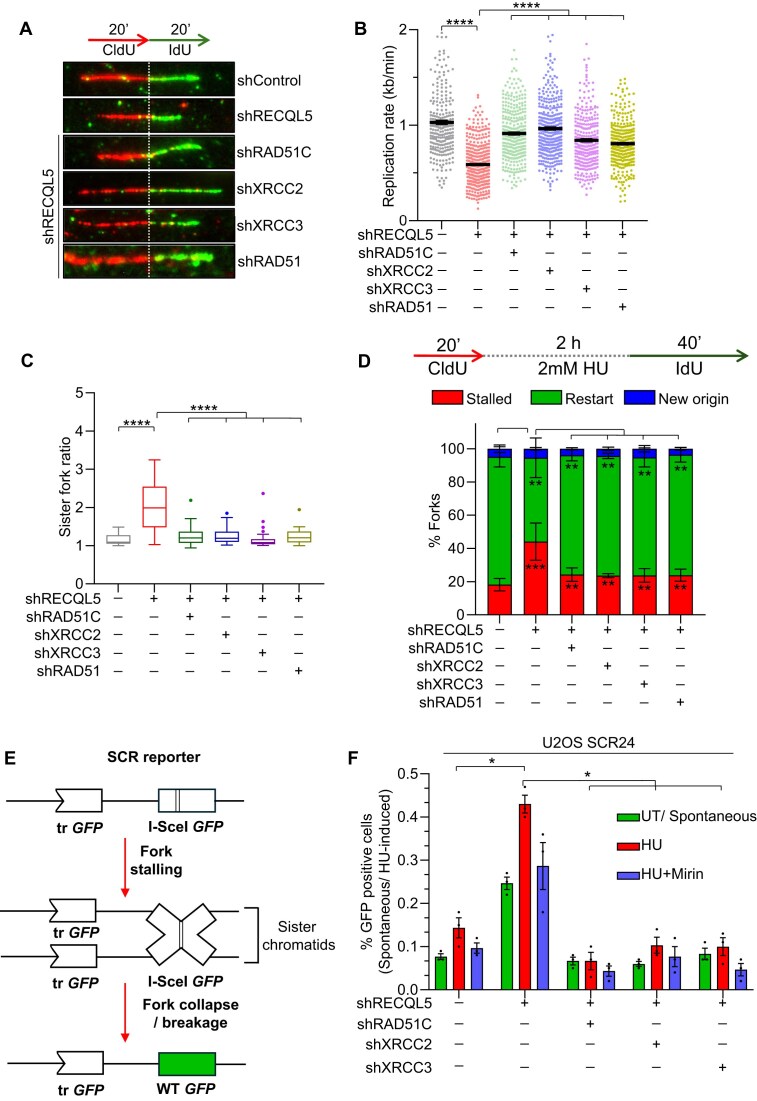
Co-depletion of HR factors restores replication defects in RECQL5-deficient cells. (**A**) (Top) Schematic of labelling scheme used for DNA fibre assay. (Bottom) Representative DNA fibre images showing ongoing replication in RECQL5 and RECQL5 co-depleted with indicated HR factors in U2OS cells. (**B**) Quantification of IdU track lengths as replication rate (kb/min) for indicated conditions shown in panel (A). A total of ≥300 DNA fibres were analysed across three biological replicates. Data are represented as mean ± SEM. Mann–Whitney *t*-test, *P< .05; **P< .001; ***P< .0001; ****P< .0001; ns, non-significant. (**C**) Quantification of fork asymmetry in U2OS cells with indicated conditions. A total of ≥35 DNA fibres emanating from a single origin were analysed across three biological replicates. Data are represented as mean ± SEM. Mann–Whitney *t*-test, *P< .05; **P< .001; ***P< .0001; ****P< .0001; ns, non-significant. (**D**) (Top) Schematic of labelling protocol used for scoring fork restart events by DNA fibre assay. (Bottom) Bar plot showing the percentage of stalled, restarted forks and new origin firing events in indicated U2OS cells. A total of ≥300 DNA fibres were analysed across three biological replicates. Data are represented as mean ± SEM. One-way ANOVA test, *P< .05; **P< .001; ***P< .0001; ****P< .0001; ns, non-significant. (**E**) A schematic of SCR depicting the mechanism of replication-born recombination events upon acute replication stress induced by a low dose of HU. (**F**) Quantification of spontaneous and HU-induced recombination frequencies in RECQL5 and RECQL5 co-depleted with indicated HR factors in U2OS SCR24 cells. Data are represented as mean ± SEM from three independent experiments. One-way ANOVA test, *P< .05; **P< .001; ***P< .0001; ****P< .0001; ns, non-significant.

Elevated RAD51 activity resulted in a decrease in the replication rate in RECQL5-depleted cells (Fig. 2A and B). Hyper-recombination at the replication forks has been shown to cause fork slowdown [50]. Hence, we asked whether hyper-recombination mediated by RAD51 might be responsible for fork slowdown in RECQL5 deficiency. To test this, we co-depleted RAD51 and RAD51 paralogues—RAD51C, XRCC2, and XRCC3—in the RECQL5-depleted cells (Supplementary Figs S3A and S4A–C) and performed DNA fibre analysis in U2OS and hTERT-RPE1 cells. Interestingly, co-depletion of RAD51 and RAD51 paralogues led to a significant rescue in the replication rate and fork asymmetry observed in RECQL5-depleted cells (Fig. 3A–C and Supplementary Fig. S4D). Furthermore, co-depletion of RAD51 and RAD51 paralogues also rescued the fork restart defects in RECQL5-depleted cells (Fig. 3D and Supplementary Fig. S4E).

We previously reported that RAD51-mediated DSB repair relies on XRCC3 phosphorylation but not on XRCC2 phosphorylation [66, 67]. To test the effect of XRCC3 phosphorylation on replication kinetics in RECQL5-depleted cells, we expressed shRNA-resistant WT and S225A XRCC3 in the background of RECQL5–XRCC3 co-depletion and performed the DNA fibre assay. Strikingly, the reduced replication rate observed in RECQL5-deficient cells was rescued by HR-deficient S225A compared to WT XRCC3 (Supplementary Fig. S5A and B). In contrast, ectopic expression of HR-proficient S247A XRCC2 [68] could not restore the replication defects in the RECQL5–XRCC2 co-depleted cells (Supplementary Fig. S5C and D). These results suggest that the decrease in replication rate in the absence of RECQL5 is a direct consequence of unregulated HR at replication forks.

RECQL5 helicase disrupts RAD51 from pre-synaptic nucleofilament [37, 69] and promotes the SDSA pathway of DSB repair [70]. Previous reports have shown that RECQL5 deficiency leads to elevated levels of HR [37] and crossover recombination [36]. Co-depletion of HR factors may rescue the hyper-recombination phenotype observed in RECQL5-depleted cells. We assessed the frequency of HR events using an SCR reporter upon inducing breaks with I-SceI endonuclease expression [14, 56] (Supplementary Fig. S4F). RECQL5 depletion led to a ∼3-fold increase in I-SceI-induced HR frequency (Supplementary Fig. S4G). Interestingly, co-depletion of RAD51 paralogues—RAD51C, XRCC2, and XRCC3—significantly reduced the elevated HR in RECQL5-depleted cells (Supplementary Fig. S4G). The SCR reporter can also be employed to study replication-born HR events, as reported previously [50, 71]. DNA breaks arising due to stalled/collapsed forks in the vicinity of the I-SceI site can lead to spontaneous HR events (Fig. 3E). Upon prolonged exposure to a low dose of HU-induced replication stress, RECQL5-depleted cells showed a significant increase in HR events compared to control cells (Fig. 3F). Notably, co-depleting RAD51 paralogues with RECQL5 rescued the elevated recombination frequency observed in RECQL5-depleted cells (Fig. 3F). Single-ended DSBs that arise from the collapsed forks need to undergo MRE11-dependent resection to facilitate HR-mediated recovery [72]. Indeed, inhibiting MRE11 by mirin was able to rescue HU-induced elevated HR events in RECQL5-depleted cells (Fig. 3F). These results suggest that RECQL5 mitigates aberrant HR events during DSB repair and with replication-born lesions. Collectively, our data demonstrate that RECQL5 plays a central role in maintaining an equilibrium of HR factors at stalled replication forks.

### RECQL5 deficiency leads to the persistence of HR factors at replication sites

**Figure 4.**
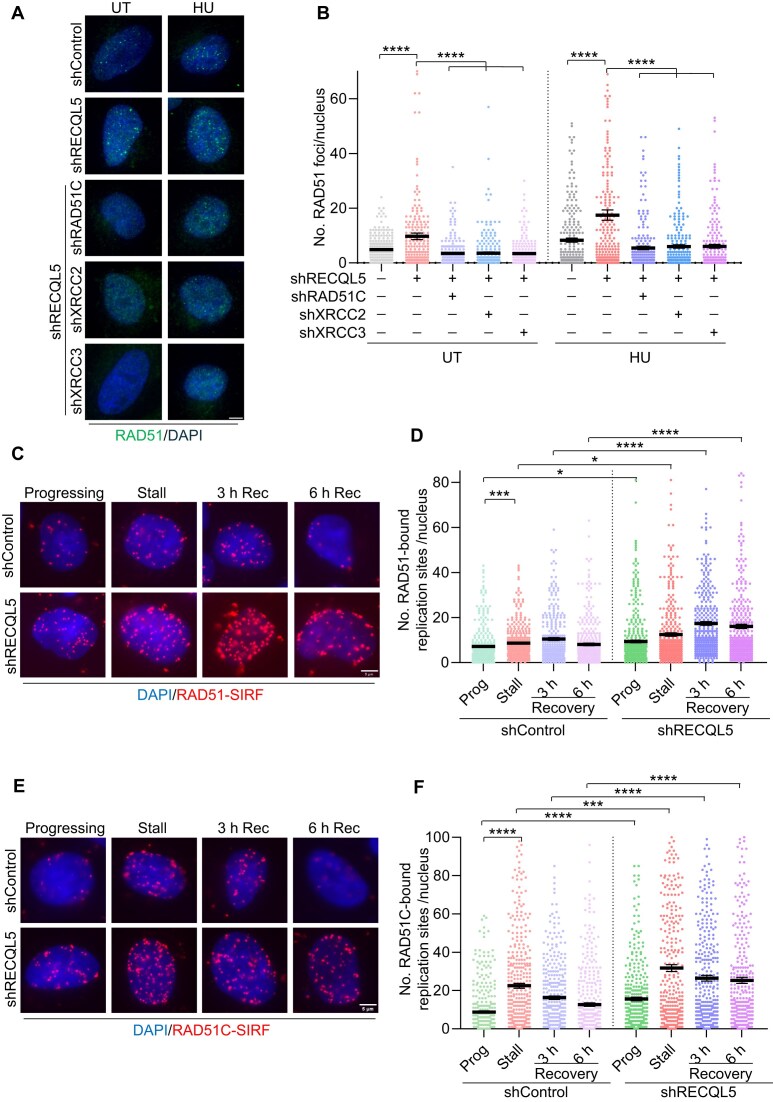
HR factors accumulate at replication sites in RECQL5-depleted cells. (**A**) Representative images of RAD51 foci in indicated U2OS cells. Cells were treated with 1 mM HU for 4 h prior to fixation, followed by immunofluorescence staining. Scale bar, 5 μm. (**B**) Quantification of RAD51 foci/nucleus with indicated conditions as shown in panel (A). A total of ≥240 nuclei were analysed across three biological replicates. Data are represented as mean ± SEM. Mann–Whitney *t*-test, *P< .05; **P< .001; ***P< .0001; ****P< .0001; ns, non-significant. (**C**) Representative images of chromatin-bound RAD51-SIRF signals at progressing forks, stalled, and recovery conditions in control and RECQL5-depleted U2OS cells. HU-treated cells were washed thrice with 1× PBS and recovered in fresh media for 3 h and 6 h to assess RAD51 persistence at stalled fork sites. Scale bar, 5 μm. (**D**) Quantification of chromatin-bound RAD51-SIRF signals as shown in panel (C). A total of ≥300 nuclei were analysed across three biological replicates. Data are represented as mean ± SEM. Mann–Whitney *t*-test, *P< .05; **P< .001; ***P< .0001; ****P< .0001; ns, non-significant. (**E**) Representative images of chromatin-bound RAD51C SIRF signals at progressing forks, stalled, and recovery conditions in control and RECQL5-depleted U2OS cells. HU-treated cells were washed thrice with 1× PBS and recovered in fresh media for 3 h and 6 h to assess RAD51C persistence at stalled fork sites. Scale bar, 5 μm. (**F**) Quantification of chromatin-bound RAD51C-SIRF signals as shown in panel (E). A total of ≥300 nuclei were analysed across three biological replicates. Data are represented as mean ± SEM. Mann–Whitney *t*-test, *P< .05; **P< .001; ***P< .0001; ****P< .0001; ns, non-significant.

RAD51 is the master regulator of HR in mammalian cells [73, 74]. We observed elevated replication-associated HR frequency in RECQL5-depleted cells (Fig. 3F). Since RECQL5 is present at actively replicating sites (Fig. 1B and C), we investigated the dynamics of RAD51 localization in the control and RECQL5-depleted cells. Strikingly, we found an increase in RAD51 foci formation upon RECQL5 depletion in unchallenged conditions, and the RAD51 foci were further elevated with HU-induced replication stress (Fig. 4A and B). Co-depletion of RAD51 paralogues—RAD51C, XRCC2, and XRCC3—in RECQL5-depleted cells rescued the elevated levels of RAD51 foci in both unchallenged and HU-induced replication stress conditions (Fig. 4A and B). To corroborate these findings, we performed SIRF analysis measuring RAD51 and RAD51 mediators in a RECQL5-depleted background with and without replication stress. Chromatin-bound RAD51, RAD51C, and XRCC3-SIRF signals were significantly elevated with and without exposure to HU in RECQL5-deficient cells (Fig. 4C–F and Supplementary Fig. S5E and F). However, upon recovery from HU stress, RAD51 and RAD51 paralogues accumulation declined gradually in control cells but persisted in RECQL5-depleted cells (Fig. 4C–F and Supplementary Fig. S5E and F). These observations demonstrate that replisome-associated RECQL5 regulates HR at both active and stalled replication forks to promote genome duplication.

Unregulated HR activity at the stalled fork sites in the absence of RECQL5 could lead to genome instability. To assess this, we investigated the status of DNA damage in RECQL5 alone and in RECQL5 and HR factors co-depleted cells. Depletion of RECQL5 led to an increase in γH2AX levels in unperturbed conditions (Supplementary Fig. S6A and B). However, co-depletion with RAD51 and RAD51C further increased the γH2AX levels (Supplementary Fig. S6A and B). Unresolved replication intermediates can lead to the formation of micronucleation and chromosomal abnormalities [1]. Depletion of RECQL5 in the background of RAD51 and RAD51C showed a modest increase in micronucleated cells (Supplementary Fig. S6C). Chromosomal abnormalities were increased in RECQL5-depleted cells upon exposure to HU (Supplementary Fig. S6D and E) and were further increased with RAD51 and RAD51C co-depletion (Supplementary Fig. S6D and E). Replication stress leads to the uncoupling of polymerase and helicase, exposing a long stretch of ssDNA. Analysis of ssDNA by native BrdU staining showed an increase in BrdU foci in RECQL5-depleted cells (Supplementary Fig. S6F). Notably, the co-depletion of RAD51 and RAD51C in the background of RECQL5-depletion led to a further increase in ssDNA (Supplementary Fig. S6F). However, although individual depletion of RECQL5 or RAD51C showed a significant decrease in survival in response to HU-induced replication stress, combined depletion did not show a further defect in survival (Supplementary Fig. S6G). Collectively, our data indicate that RECQL5 is necessary for genome maintenance by regulating HR factors upon replication stress.

### The RAD51 interaction and the helicase activity of RECQL5 are essential for facilitating genome-wide replication

**Figure 5.**
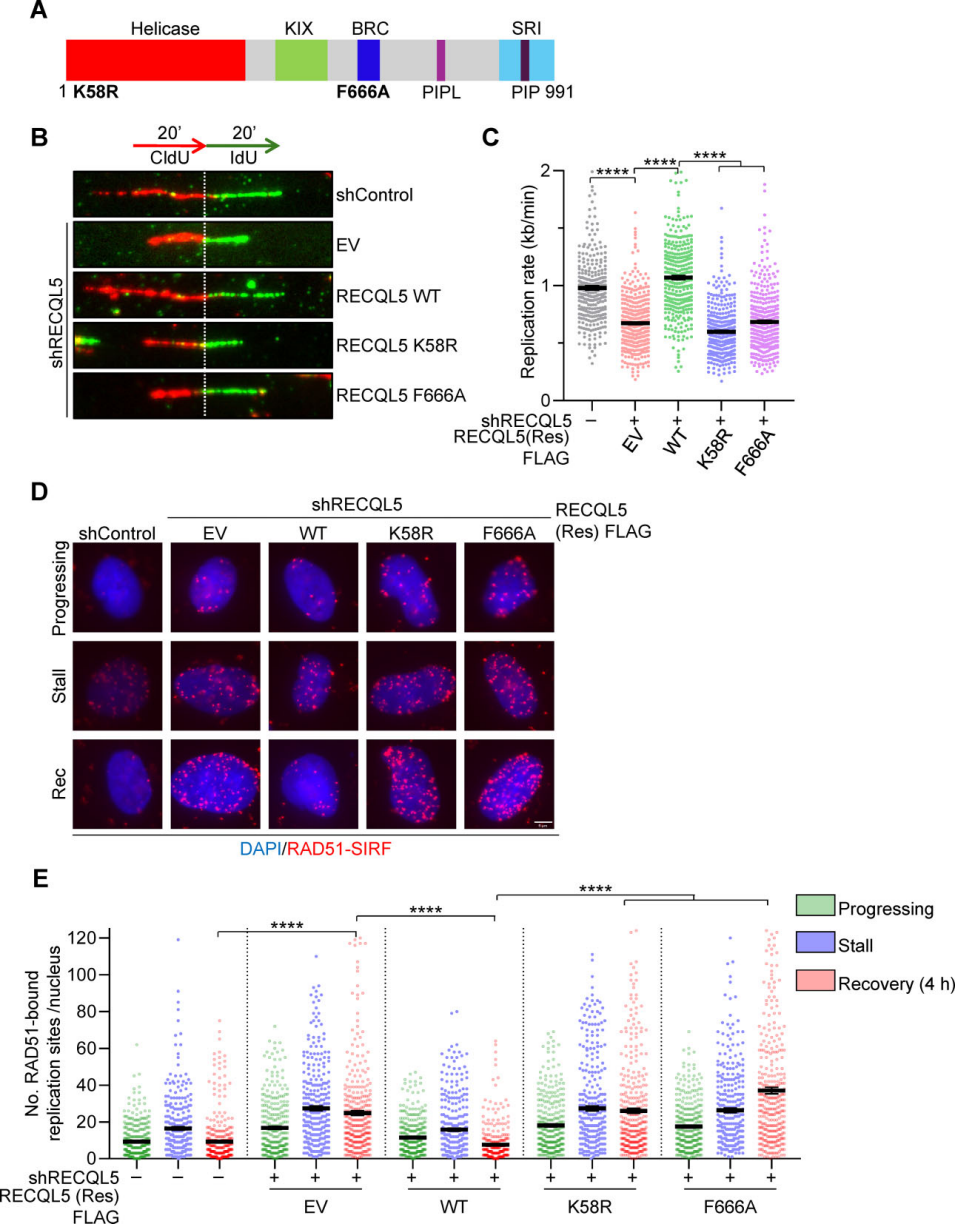
RECQL5 helicase activity and its interaction with RAD51 are essential for genome-wide replication. (**A**) Domain architecture of RECQL5 helicase. Sites of point mutations in helicase (K58R) and BRC domain (F666A) are indicated. (**B**) (Top) Schematic of labelling scheme used for DNA fibre assay. (Bottom) Representative DNA fibre images to show the replication rate in the indicated U2OS cells. (**C**) Quantification of IdU track lengths as replication rate (kb/min) as shown in panel (B). A total of ≥300 DNA fibres were analysed across three biological replicates. Data are represented as mean ± SEM. Mann–Whitney *t*-test, *P< .05; **P< .001; ***P< .0001; ****P< .0001; ns, non-significant. (**D**) Representative images of chromatin-bound RAD51-SIRF signals at progressing, stalled, and recovering forks in indicated U2OS cells. HU-treated cells were washed thrice with 1× PBS and recovered in fresh media for 4 h to assess RAD51 persisting at stalled fork sites. Scale bar, 5 μm. (**E**) Quantification of chromatin-bound RAD51-SIRF signals as indicated in panel (D). A total of ≥300 nuclei were analysed across three biological replicates. Data are represented as mean ± SEM. Mann–Whitney *t*-test, *P< .05; **P< .001; ***P< .0001; ****P< .0001; ns, non-significant.

RECQL5 possesses a helicase domain at the N-terminus and this domain is important for its ssDNA translocation in 3′ to 5′ direction [69, 75]. To test whether the helicase activity of RECQL5 is required for restricting RAD51-mediated excessive fork remodelling to promote DNA replication, we generated an shRNA-resistant helicase-dead K58R RECQL5 mutant (Fig. 5A). Expression of WT RECQL5 but not K58R mutant rescued replication defects in RECQL5-deficient cells (Fig. 5B, 5C, and Supplementary Figs. S7A–C), suggesting that helicase activity of RECQL5 is essential for regulating RAD51 during DNA replication. RECQL5 physically interacts with RAD51 via the BRC domain and disrupts RPA, RAD51, and DMC1 from ssDNA [69]. A point mutation introduced in the BRC domain, the F666A (Fig. 5A), disrupts the RECQL5–RAD51 interaction and affects its RAD51-stripping ability [38]. To test whether RECQL5 interaction with RAD51 is crucial for limiting the RAD51 activity at stalled fork sites, we expressed shRNA-resistant F666A RECQL5 mutant in RECQL5-depleted cells and measured the replication rate. In comparison to WT, the F666A RECQL5 mutant failed to rescue the replication defect in RECQL5-depleted cells, indicating that RECQL5 interaction with RAD51 is required for suppressing the aberrant activity of RAD51 at stalled fork sites (Fig. 5B, 5C, and Supplementary Figs. S7A–C).

Electron microscopy analysis showed that the expression of K58R and F666A mutants in the RECQL5-depleted cells resulted in an increase in the accumulation of reversed forks in unperturbed conditions [49]. This could be due to excessive RAD51 accumulation at forks. To test this, we measured RAD51 accumulation at the stalled fork sites by performing a RAD51-SIRF assay. Consistent with our previous observations (Fig. 4C and D), RAD51 accumulates at active and stalled forks and persists during recovery from HU-induced replication stress in RECQL5-depleted cells (Fig. 5D and E). Expression of WT RECQL5 rescued this phenotype (Fig. 5D and E), but K58R and F666A RECQL5 mutants failed to restrict RAD51 accumulation at the fork sites (Fig. 5D and E). A previous study demonstrated that phosphorylation of RECQL5 at S727 by CDK1 is required to regulate RAD51 activity to promote MiDAS at CFSs [48]. To test whether RECQL5 phosphorylation is required for genome-wide replication, we generated a phospho-dead RECQL5-S727A mutant and performed a DNA fibre assay. Notably, expression of shRNA-resistant RECQL5-S727A (Supplementary Fig. S7D) rescued the replication defects observed upon RECQL5 depletion (Supplementary Fig. S7E), suggesting that RECQL5-S727 phosphorylation is dispensable for replication fork progression. Collectively, these observations indicate that the RAD51 interaction and the helicase activity of RECQL5, but not its phosphorylation, are critical for limiting the RAD51 activity at the fork sites to promote genome duplication.

### RECQL5 and RTEL1 participate in the same pathway to regulate RAD51 at the fork sites

**Figure 6.**
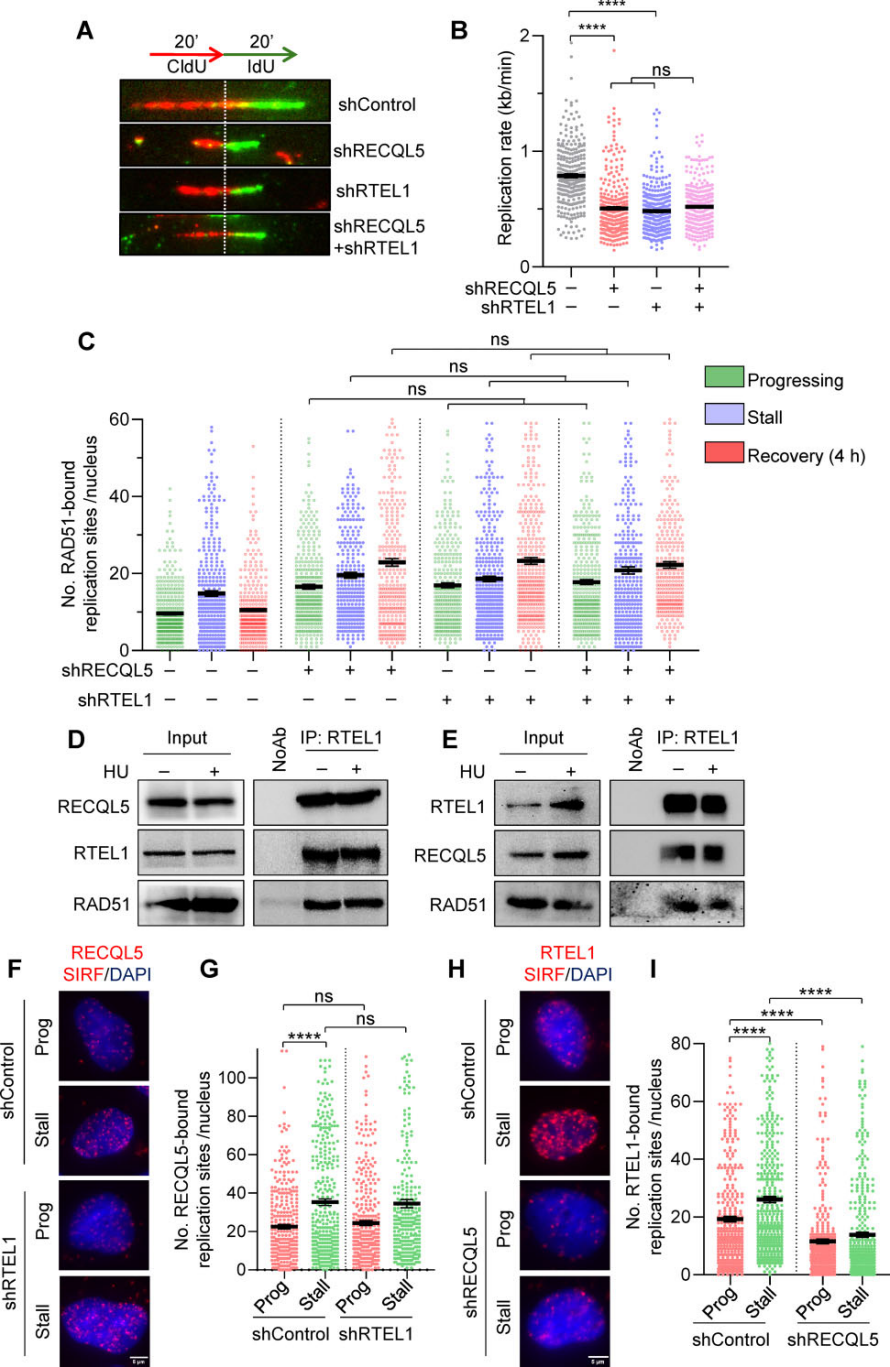
RECQL5 functions upstream of RTEL1 in regulating RAD51-mediated fork reversal. (**A**) (Top) Schematic of labelling used for DNA fibre assay. (Bottom) Representative DNA fibre images to show fork slowdown in indicated U2OS cells. (**B**) Quantification of IdU track lengths as replication rate (kb/min) in cells as shown in panel (A). A total of ≥300 DNA fibres were analysed across three biological replicates. Data are represented as mean ± SEM. Mann–Whitney *t*-test, *P< .05; **P< .001; ***P< .0001; ****P< .0001; ns, non-significant. (**C**) Quantification of chromatin-bound RAD51-SIRF signals in the indicated U2OS cells. HU-treated cells were washed thrice with 1× PBS and recovered in fresh media for 4 h to assess RAD51 persisting at stalled fork sites. A total of ≥300 nuclei were analysed across three biological replicates. Data are represented as mean ± SEM. Mann–Whitney *t*-test, *P< .05; **P< .001; ***P< .0001; ****P< .0001; ns, non-significant. (**D**) Co-IP of endogenous RECQL5 from U2OS cells with or without HU treatment (2 mM, 4 h), followed by immunoblotting with indicated antibodies. (**E**) Co-IP of endogenous RTEL1 from U2OS cells with or without HU treatment (2 mM, 4 h), followed by immunoblotting with indicated antibodies. (**F**) Representative images of chromatin-bound RECQL5-SIRF signals at progressing and stalled conditions in control and RTEL1-depleted U2OS cells. Scale bar, 5 μm. (**G**) Quantification of chromatin-bound RECQL5-SIRF signals as shown in panel (F). A total of ≥300 nuclei were analysed across three biological replicates. Data are represented as mean ± SEM. Mann–Whitney *t*-test, *P< .05; **P< .001; ***P< .0001; ****P< .0001; ns, non-significant. (**H**) Representative images of chromatin-bound RTEL1-SIRF signals at progressing and stalled conditions in control and RECQL5-depleted U2OS cells. Scale bar, 5 μm. (**I**) Quantification of chromatin-bound RTEL1-SIRF signals as shown in panel (H). A total of ≥300 nuclei were analysed across three biological replicates. Data are represented as mean ± SEM. Mann–Whitney *t*-test, *P< .05; **P< .001; ***P< .0001; ****P< .0001; ns, non-significant.

Our previous study demonstrates that the RTEL1 helicase counteracts RAD51-mediated excessive fork remodelling to facilitate genome duplication [50]. To test whether RECQL5 and RTEL1 function independently or co-ordinate in a common pathway to restrict RAD51-mediated excessive fork reversal, we measured DNA replication in RECQL5 or RTEL1 alone and RTEL1-RECQL5 co-depleted cells. Individual depletions of RECQL5 or RTEL1 showed a decrease in replication rate (Fig. 6A, 6B, and Supplementary Fig. S7F). Notably, co-depletion of RECQL5 with RTEL1 did not show any further reduction compared to RECQL5 or RTEL1 alone depleted cells (Fig. 6A, 6B, and Supplementary Fig. S7F). We further measured RAD51 accumulation at stalled forks upon co-depletion of RECQL5 with RTEL1 by performing a RAD51-SIRF assay. Depletion of either RECQL5 or RTEL1 resulted in accumulation of RAD51 at active and stalled forks and persisted after recovery from HU-induced replication stress (Fig. 6C and Supplementary Fig. S7G). However, co-depletion of both RECQL5 and RTEL1 did not show any additional increase in RAD51 accumulation (Fig. 6C and Supplementary Fig. S7G). These results indicate that RECQL5 and RTEL1 are epistatic in regulating aberrant RAD51 activity at stalled fork sites.

To understand the mechanism by which RECQL5 and RTEL1 co-ordinate to regulate RAD51-mediated fork reversal, we examined whether they interact with each other. Co-IP using RECQL5 antibody revealed an interaction of RECQL5 with RTEL1 in control as well as HU-treated cells (Fig. 6D). The RAD51, which is known to interact with RECQL5 [38], served as a positive control (Fig. 6D). To confirm these observations, we performed reciprocal-IP using RTEL1 antibody and found interactions with RECQL5 and RAD51 in control and HU-treated cells (Fig. 6E). The Co-IP experiments were performed in the absence of benzonase enzyme, and hence the interactions observed with RECQL5 and RTEL1 could also be mediated by DNA. However, these results prompted us to ask whether RECQL5 localization to fork sites is dependent on RTEL1. Our analysis of RECQL5-SIRF foci in RTEL1-depleted cells showed that the localization of RECQL5 at the active and stalled forks remained unchanged compared to control cells (Fig. 6F and G). Interestingly, a significant reduction in RTEL1-SIRF foci was observed upon RECQL5 depletion at the active and stalled forks compared to control cells (Fig. 6H and I). These data suggest that RECQL5 functions upstream of RTEL1 in regulating RAD51 hyperactivity at stalled fork sites.

### RECQL5-mediated regulation of RAD51 and transcription elongation is separable

**Figure 7.**
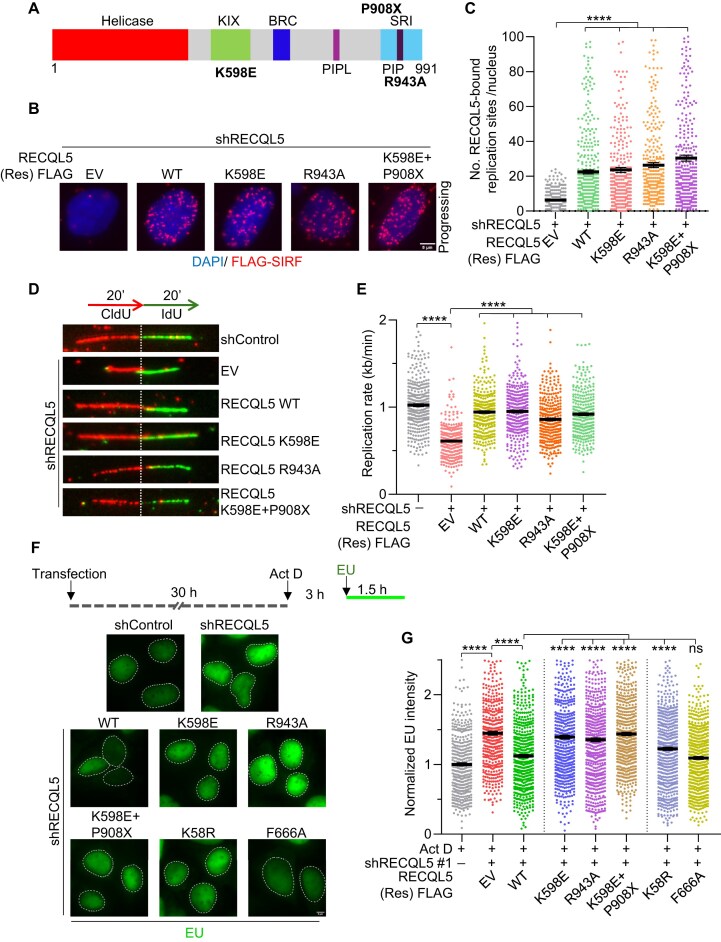
Distinct functions of RECQL5 in regulating DNA replication and transcription elongation. (**A**) Domain architecture of RECQL5 helicase. Sites of point mutations in the KIX domain (K598E), SRI domain (R943A), and SRI domain deletion (P908X) are indicated. (**B**) Representative images of chromatin-bound RECQL5-FLAG SIRF signals at active replication sites in the indicated U2OS cells. Scale bar, 5 μm. (**C**) Quantification of chromatin-bound RECQL5-FLAG SIRF signals as shown in panel (B). A total of ≥300 nuclei were analysed across three biological replicates. Data are represented as mean ± SEM. Mann–Whitney *t*-test, *P< .05; **P< .001; ***P< .0001; ****P< .0001; ns, non-significant. (**D**) (Top) Schematic of labelling scheme used for DNA fibre assay. (Bottom) Representative DNA fibre images to show the replication rate in the indicated U2OS cells. (**E**) Quantification of IdU track lengths as replication rate (kb/min) as shown in panel (D). A total of ≥300 DNA fibres were analysed across three biological replicates. Data are represented as mean ± SEM. Mann–Whitney *t*-test, *P< .05; **P< .001; ***P< .0001; ****P< .0001; ns, non-significant. (**F**) (Top) Schematic of the experimental workflow of transcription elongation assay. (Bottom) Representative images of the intensity of EU incorporation in the indicated U2OS cells. Cells were treated with 0.05 μg/ml actinomycin D (RNAPI inhibitor) for 3 h. One millimolar EU was added to cells for 1.5 h prior to the endpoint. Nuclear boundaries are marked with white-dashed lines. Scale bar, 5 μm. (**G**) Quantification of EU intensities normalized to control as indicated in panel (F). A total of ≥450 nuclear intensities were analysed across three biological replicates. Data are represented as mean ± SEM. Mann–Whitney *t*-test, *P< .05; **P< .001; ***P< .0001; ****P< .0001; ns, non-significant.

RECQL5 is the only RecQ family helicase identified to interact with RNAPII and regulate transcription through a unique C-terminal domain (CTD), which is absent in other RecQ helicases [33, 34]. This C-terminal extension harbours the KIX and SRI domains, which are essential for RECQL5 interaction with RNAPII to inhibit transcription and prevent transcription-associated genome instability [40, 58, 76]. The SRI domain binds specifically to the phosphorylated CTD of RNAPII, while the KIX domain competes with the transcription elongation factor TFIIS to inhibit transcription [41]. To test whether the transcription and replication regulatory functions of RECQL5 are independent of each other, we introduced previously reported point mutations, K598E in the KIX domain [41] and R943A in the SRI domain [58, 77], into RECQL5, which abrogates its interaction with RNAPII (Fig. 7A). Using the SIRF assay, we first evaluated whether these mutants were recruited to active forks. Expression of the shRNA-resistant K598E and R943A RECQL5 mutants in the RECQL5-depletion background (Supplementary Fig. S8A) showed similar levels of RECQL5-FLAG-SIRF foci compared to WT RECQL5-expressing cells (Fig. 7B and C). The C-terminal end of RECQL5 also has been shown to interact with RNAPI and regulate TRCs at ribosomal DNA arrays [47]. We generated a C-terminal truncation mutant P908X in the K598E mutant background to abolish RECQL5 interaction with both RNAPI and RNAPII (Fig. 7A). Notably, FLAG-RECQL5-SIRF foci observed with K598E + P908X RECQL5-expressing cells were comparable to WT RECQL5-expressing cells (Fig. 7B, 7C, and Supplementary Fig. S8A). Remarkably, the expression of K598E, R943A, or K598E + P908X RECQL5 in the RECQL5-depletion background showed no defect in the replication rate compared to control cells (Fig. 7D and E), suggesting that RECQL5 interaction with RNAPI and RNAPII is dispensable for its RAD51 regulation during replication.

The KIX and SRI domain mutants have been shown to regulate transcription elongation rate in *in vitro* assay systems [40, 41]. RECQL5 depletion has been linked to a global increase in transcription rate and transcription-associated genome instability [43, 56]. We, therefore, sought to determine if there is any crosstalk between the replication and transcription elongation regulation by RECQL5. To test this, we adopted a recently developed assay that specifically measures the RNAPII elongation rate at a single-cell resolution [55]. We pulse labelled U2OS cells with 5-Ethynyl uridine (EU) to label newly synthesized RNA in the presence of a very low dose of actinomycin D (Fig. 7F). Actinomycin D is a specific inhibitor of RNAPI, thereby allowing EU incorporation specifically through RNAPII. EU is then conjugated to a fluorophore by a click reaction, and fluorescence intensity serves as a measure of RNAPII-mediated transcription activity in the cells [55]. We expressed RNAPI/II interaction mutants K598E, R943A, K598E + P908X of RECQL5, and the helicase K58R, and RAD51 interaction-deficient F666A RECQL5 mutants in the background of RECQL5-depletion (Supplementary Fig. S8B) and performed the transcription elongation assay. The depletion of RECQL5 resulted in an increase in EU intensity compared to control cells, validating RECQL5 role in transcription regulation (Fig. 7F and G). Notably, the expression of RNAPI/II interaction-deficient mutants K598E, R943A, and K598E + P908X also led to an increase in EU intensity compared to WT RECQL5-expressing cells (Fig. 7F and G). Interestingly, the expression of helicase dead mutant K58R led to an increase in EU intensity compared to WT RECQL5-expressing cells (Fig. 7F and G). An increase in transcriptional activity in the RECQL5 helicase-dead mutant further supports the earlier reported finding that the RECQL5 helicase domain is required to engage with the DNA template for its transcription regulation [41, 78–79]. Strikingly, the RECQL5–RAD51 interaction mutant (F666A), which was defective in suppressing excessive fork reversal by RAD51, was competent for transcription regulation (Fig. 7F and G), suggesting that regulation of RAD51-mediated fork remodelling and transcriptional elongation functions are mutually exclusive. These results demonstrate that RECQL5 regulates DNA replication and transcription elongation by independent mechanisms.

### Abrogation of RECQL5-mediated RAD51 and transcription regulation leads to elevated genome instability

**Figure 8.**
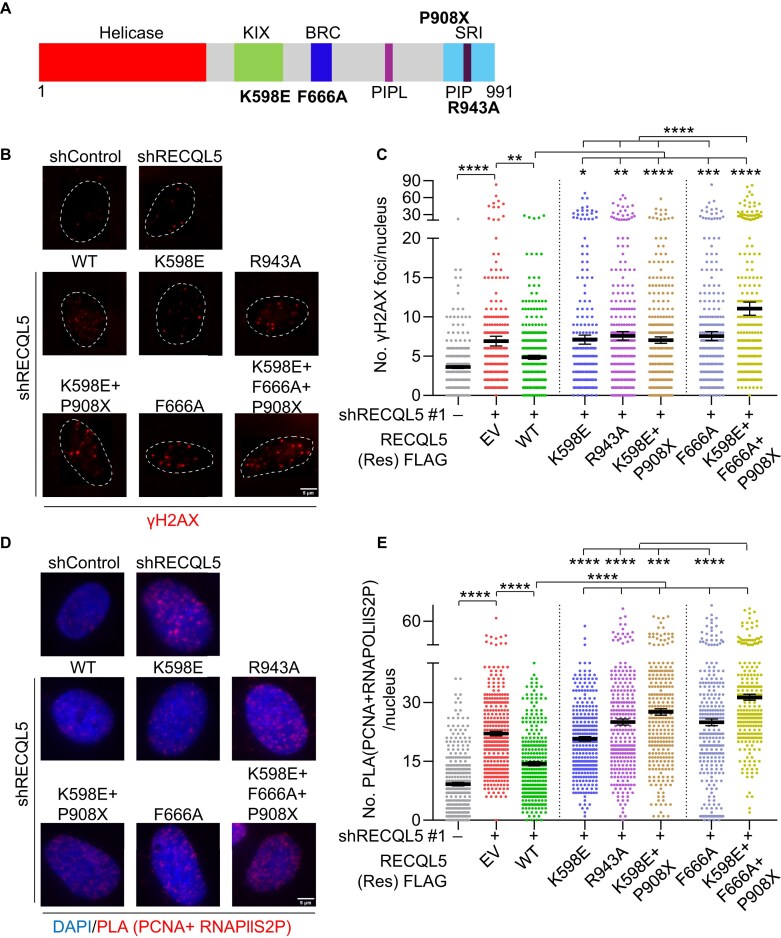
Both DNA replication and transcription elongation functions of RECQL5 are essential for genome stability. (**A**) Domain architecture of RECQL5 helicase. Sites of point mutations in the KIX domain (K598E), SRI domain (R943A), BRC domain (F666A), and SRI domain deletion (P908X) are indicated. (**B**) Representative images of chromatin-bound γH2AX foci in indicated U2OS cells. Nuclear boundaries are marked with white-dashed lines. Scale bar, 5 μm. (**C**) Quantification of chromatin-bound γH2AX foci/nucleus for conditions as shown in panel (B). A total of ≥300 nuclei were analysed across three biological replicates. Data are represented as mean ± SEM. Mann–Whitney *t*-test, *P< .05; **P< .001; ***P< .0001; ****P< .0001; ns, non-significant. (**D**) Representative images of PCNA + RNAPIIS2P PLA foci in the indicated U2OS cells. Scale bar, 5 μm. (**E**) Quantification of PCNA + RNAPIIS2P PLA foci/nucleus for conditions as shown in panel (D). A total of ≥300 nuclei were analysed across three biological replicates. Data are represented as mean ± SEM. Mann–Whitney *t*-test, *P< .05; **P< .001; ***P< .0001; ****P< .0001; ns, non-significant.

Since RECQL5 regulates transcription elongation and HR during DNA replication by independent mechanisms, we next examined the effect of genome stability when both functions are abrogated. For this, we introduced an F666A mutation in the K598E + P908X mutant background (Fig. 8A), thereby generating a mutant defective in both activities. We expressed RNAPI/II interaction mutants (K598E, R943A, K598E + R908X), RAD51 interaction-deficient (F666A), and a combined RNAPI/II and RAD51 interaction-deficient mutant (K598E + F666A + P908X) in the background of RECLQ5 depletion (Supplementary Fig. S8B) and examined DNA breaks by γH2AX foci. The depletion of RECQL5 resulted in an increase in γH2AX foci, which was rescued upon expression of WT-RECQL5. Notably, the expression of either RNAPI/II interaction-deficient mutants (K598E, R943A, and K598E + P908X) or RAD51-interaction-deficient mutants (F666A) led to an increase in γH2AX compared to WT-RECQL5-expressing cells (Fig. 8B and C). Strikingly, expression of combined RNAPI/II- and RAD51-interaction-deficient mutant (K598E + F666A + P908X), which is defective in suppressing fork reversal and regulating transcription, led to a further increase in γH2AX foci compared to RNAPI/II-interaction-deficient (K598E, R943A, and K598E + P908X) alone or RAD51-interaction-deficient (F666A) alone mutants (Fig. 8B and C). TRCs are the major source of genome instability when either normal transcription or replication is perturbed [43, 80]. Accumulation of DNA breaks could be due to increased TRCs in the cells. To test this, we performed a PLA between elongating RNA polymerase II (RNAPII S2P) and PCNA. The depletion of RECQL5 led to a significant increase in TRCs compared to control cells. The expression of either RNAPI/II interaction-deficient mutants (K598E, R943A, and K598E + P908X) or RAD51-interaction-deficient mutants (F666A) also led to an increase in TRCs compared to WT RECQL5-expressing cells (Fig. 8D and E). Interestingly, expression of the combined RNAPI/II and RAD51 interaction-deficient mutant (K598E + F666A + P908X) caused a further increase in TRCs (Fig. 8D and E), indicating an elevated genome instability when both the RECQL5 functions are compromised. Collectively, our findings demonstrate that RECQL5 safeguards genome stability through its distinct functions in regulating transcription elongation and RAD51-mediated fork reversal.

## Discussion

The RecQ family of genes in humans is known to have multiple functions in genome maintenance and the prevention of tumorigenesis [35]. Mice lacking Recql5 are predisposed to cancer and exhibit a high rate of HR and elevated chromosomal rearrangements in response to replication stress [37]. Notably, mutations in RECQL5 are known to be associated with various types of cancers, including breast, osteosarcoma, and head and neck cancer [32, 81–83], suggesting the crucial role of RECQL5 in genome maintenance and tumour suppression. Despite the identification of RECQL5 roles in HR regulation, DNA replication, transcription elongation, and resolution of TRCs [33, 34, 84], the precise mechanism by which RECQL5 controls multiple functions in genome maintenance is obscure. Data presented here demonstrate that RECQL5 distinctly regulates RAD51-mediated fork reversal and transcriptional elongation to maintain genome integrity.

**Figure 9.**
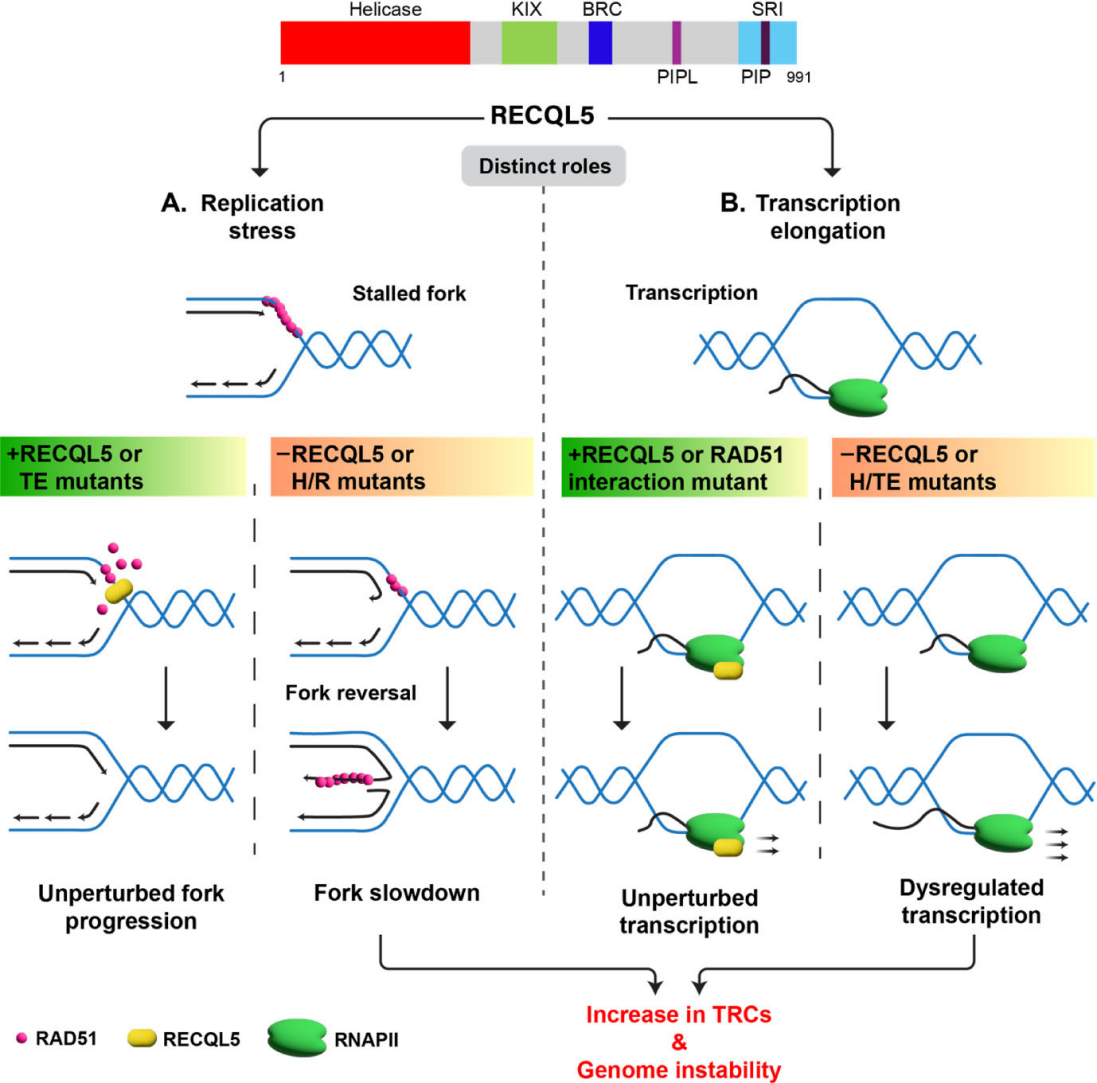
A model illustrating the distinct roles of RECQL5 in regulating RAD51-mediated fork reversal during DNA replication and in transcription elongation. (**A**) RECQL5 strips RAD51 from stalled replication forks, thereby inhibiting RAD51-mediated fork reversal in a manner dependent on its helicase activity/RAD51-interaction (H/R). The mutants that are devoid of their ability to regulate transcription elongation (TE) are competent in stripping RAD51 from stalled forks. (**B**) RECQL5 inhibits transcription elongation through its interaction with RNAPII. The RAD51 interaction mutant remains competent in regulating transcription. Loss of both transcription elongation and RAD51-mediated fork reversal activities of RECQL5 leads to elevated levels of TRCs and genome instability.

RECQL5 associates at the active replication sites and is enriched at the stalled fork sites upon inducing replication stress with HU. Its enrichment at the stalled fork sites is dependent on the PIPL motif but not the PIP motif. RECQL5 depletion leads to a reduction in global DNA replication, and unrestrained DNA synthesis requires RECQL5 interaction with PCNA via the PIPL motif. Stalled forks undergo remodelling by RAD51-mediated fork reversal and fork remodelling factors [5, 63, 64, 85]. This response slows down DNA replication, prevents nucleolytic degradation of stalled forks, and facilitates restart of stalled forks [3, 4]. The replication defect in RECQL5-deficient cells can be rescued by co-depletion of RAD51 and RAD51 paralogues as well as fork remodelling factors SMARCAL1, ZRANB3, HLTF, and FBH1. Moreover, RAD51 and RAD51C/XRCC3 persist at the stalled fork sites in the RECQL5-depleted cells. These data suggest that RECQL5 restricts RAD51-mediated excessive fork reversal to promote genome duplication. Consistently, purified RECQL5 has been shown to dismantle RAD51 filaments from ssDNA [37, 38]. It is likely that RECQL5 similarly disassembles RAD51 from the sites of stalled forks to promote unrestrained DNA synthesis (Fig. 9). Indeed, it has been shown that RECQL5-mediated RAD51 disruption at the stalled forks promotes replication restart from the R-loop sites and MUS81-dependent MiDAS at CFSs [48, 49].

Remodelled forks also facilitate the restart of stalled forks by RAD51-mediated recombinase function [19, 86]. In the absence of RECQL5, uncontrolled RAD51-mediated fork reversal and HR activity at the stalled replication sites might impede global DNA replication. In fact, RECQL5-deficient cells showed elevated HU-induced HR events, and this was rescued by co-depletion of RAD51 paralogues. Moreover, the expression of HR-defective RAD51 mutants also rescued replication defects in RECQL5-depleted cells. Notably, RECQL5 ATPase and RAD51 interaction mutants were defective in rescuing replication defects in RECQL5-deficient cells. These data suggest that RECQL5 prevents RAD51-mediated excessive fork reversal and HR during DNA replication in a manner dependent on its helicase activity and interaction with RAD51 (Fig. 9). Consistently, *in vitro* studies have demonstrated that RECQL5-mediated RAD51 disassembly requires its helicase activity and interaction with RAD51 [37, 38].

Our data with HR/SCR reporter show a 3–4-fold increase in both spontaneous and HU-induced HR events in RECQL5-depleted cells. Stalled forks that are not repaired or restarted are susceptible to breakage by MUS81 or SLX1/SLX4 nucleases, leading to collapsing of forks into one-ended DSBs [87]. Such breaks could undergo processing by MRE11 and subsequently be repaired by the RAD51-mediated recombination mechanism [9, 86–88]. Indeed, inhibition of MRE11 by mirin rescued HU-induced hyper-recombination at the stalled fork sites in RECQL5-deficient cells. In addition, co-depletion of RAD51 paralogues rescued elevated HR and replication defects in the RECQL5-depleted cells. These data suggest that RECQL5 is required to suppress RAD51-mediated fork remodelling and hyper-recombination at the stalled fork sites to promote error-free genome duplication (Fig. 9). RAD51 has also been implicated in facilitating the restart of replication from the reversed forks without generating DSBs. In this context, RAD51 filaments from the reversed forks invade the template DNA ahead of the fork to generate a D-loop to facilitate fork repair and restart [9, 19]. However, the HR/SCR reporter that we are using doesn’t exclusively provide information on HR events that occur from the reversed forks without collapsing into breaks. However, further studies are needed to understand the mechanism underlying the repair and restart of replication from the stalled forks.

RAD52 has been shown to prevent excessive fork reversal by regulating SMARCAL1 at the stalled fork sites [27]. Our previous study showed that RTEL1 helicase facilitates global DNA replication by preventing RAD51-mediated fork remodelling [50]. Interestingly, co-depletion of RTEL1 with RECQL5 did not show a further reduction in replication compared to RECQL5- or RTEL1-alone-depleted cells, suggesting that RECQL5 and RTEL1 participate in the same pathway of regulating RAD51-mediated fork remodelling. Moreover, RECQL5 appears to form a complex with RTEL1 and facilitates its localization to the stalled fork sites. The RADX protein that binds to ssDNA and RAD51 also alleviates RAD51-mediated excessive fork reversal to promote genome duplication [30, 89–91]. Nonetheless, further studies are required to understand whether an interplay exists between RECQL5, RTEL1, RAD52, and RADX in regulating RAD51-mediated fork remodelling. In addition to RECQL5, studies have shown that PARI, FBH1, and FIGNL1 also disrupt RAD51 filaments and suppress hyper-recombination during DSB repair [92–97]. However, whether PARI, FBH1, or FIGNL1 promotes global DNA replication by regulating RAD51-mediated excessive fork reversal and hyper-recombination needs further investigation.

RECQL5 also regulates transcription elongation and prevents transcription-dependent genome instability by its interaction with RNAPII via KIX and SRI domains [40, 41, 76]. It is unclear whether RECQL5 independently regulates transcription elongation and RAD51-mediated fork reversal. Strikingly, RECQL5 mutants that affect RNAPII interaction were proficient in regulating DNA replication, suggesting that RECQL5-mediated RAD51 disruption activity is independent of transcription elongation regulation. Notably, RECQL5–RAD51 interaction mutant (F666A), defective for eviction of RAD51 from the stalled fork sites, was efficient in controlling the transcription elongation rate. These data suggest that RECQL5 distinctly regulates RAD51-mediated fork remodelling and transcription elongation (Fig. 9).

RECQL5 mutations have been identified in various types of cancers [82, 83], suggesting its crucial role in genome maintenance and tumour suppression. How does a defect in RECQL5 function lead to genome instability and oncogenesis? RAD51 plays an important role in the repair of DSBs by HR and replication stress responses, thereby maintaining genome stability [16, 73, 74, 98, 99]. However, aberrant HR during DNA replication can lead to the accumulation of mutations and chromosomal rearrangements [100–103]. Unregulated RAD51-mediated fork remodelling and HR in the absence of RECQL5 can lead to genome instability. Indeed, RECQL5-depleted cells exhibited an increase in DSBs and chromosomal aberrations. Defects in the RECQL5-mediated regulation of transcription elongation have been shown to cause transcription-associated DNA breaks [76]. The chromosomal breakpoints were also associated with CFSs [43], which are prone to breakage with replication stress. In fact, RECQL5 deficiency has been shown to cause replisome stalling at actively transcribed genes [47]. Defects in the RNAPII-mediated transcription also induce replication stress by increasing the burden of TRCs [43, 104–106]. Independent studies have shown that RECQL5 promotes the resolution of TRCs [47–49, 59]. Notably, expression of RECQL5 mutant defective for suppressing RAD51-mediated fork reversal and transcription regulation resulted in elevated TRCs and genome instability compared to the RECQL5–RAD51 or RECQL5–RNAPI/II interaction mutants. The increase in the TRCs and genome instability in RECQL5-deficient cells could be due to defects in the distinct functions of RECQL5 in regulating RAD51 at the forks and transcription elongation (Fig. 9).

Cancer cells continue to replicate their genomes, tolerating high replication stress and genome instability [107–109]. Unprotected forks undergo nucleolytic degradation, and fork degradation is linked to chemosensitivity in cancer cells, whereas restoration of fork protection is associated with drug resistance [29, 107, 108]. Interestingly, stalled forks are susceptible to degradation in RECQL5-deficient cells. The absence of RECQL5 promotes RAD51-mediated excessive fork reversal, which can be exploited for therapy by targeting RECQL5 in cancer cells. RECQL5 deficiency also leads to an increase in transcription-associated genome instability and TRCs [34]. Thus, RECQL5 can be targeted to increase the burden of replication stress and TRCs to sensitize the cancer cells. PARP inhibitors are widely used for the treatment of breast and ovarian cancer cells with *BRCA1*, *BRCA2*, and other HR pathway gene mutations [107, 108, 110, 111]. RECQL5 is overexpressed in many cancers, including breast and urothelial carcinoma of the bladder, and is associated with poor prognosis and survival [82]. RECQL5 can be targeted independently or in combination with PARP inhibitors or other cancer-therapeutic drugs for the treatment of cancer cells [110, 112]. However, there are reports of cancer cells developing resistance to chemotherapeutic drugs and radiation therapy [113, 114]. RECQL5 can be a potential target for the treatment of such chemo- or radioresistant cancer cells. Indeed, recent studies showed that RECQL5 can be targeted with a small molecule to preferentially kill the RECQL5-overexpressing breast cancer cells, and this molecule sensitizes cisplatin-resistant tumour cells and is effective in combination with a PARP inhibitor [115, 116].

## Supplementary Material

gkaf1019_Supplemental_File

## Data Availability

All the data supporting this study are within the article and its online supplementary material. Reagents generated in this study are available from the corresponding author upon request.
