# Supplementary material for: Distinct roles of RECQL5 in RAD51-mediated fork reversal and transcription elongation
Source: Nucleic Acids Res. 2025 Oct 14;53(19):gkaf1019. doi: 10.1093/nar/gkaf1019 (PMC12526125; doi:10.1093/nar/gkaf1019)
Supplement: gkaf1019_Supplemental_File [file gkaf1019_supplemental_file.pdf]

Supplementary figure legends

Supplementary Figure 1

(A) (Left) Representative images of chromatin-bound RECQL5- SIRF signals in control and RECQL5 depleted U2OS cells. Scale bar, 5µm. (Right) Quantification of chromatin-bound RECQL5 SIRF. A total of ≥250 cells were analysed across two biological replicates. Data is represented as mean ±SEM. Mann-Whitney t test, *p<0.05; **p<0.001; ***p<0.0001; ****p<0.0001; ns, non-significant.

(B) (Left) Representative DNA fiber images to show fork slowdown in control and RECQL5-depleted hTERT-RPE1 cells. The schematic of the labeling scheme used for DNA fiber assay is indicated above. (Right) Quantification of IdU track lengths in control and RECQL5-depleted cells. A total of ≥225 DNA fibres were analysed across two biological replicates. Data is represented as mean ±SEM. Mann-Whitney t test, *p<0.05; **p<0.001; ***p<0.0001; ****p<0.0001; ns, non-significant.

(C) (Top) Schematic depicting symmetrical and asymmetrical bidirectional forks emanating from a single origin firing event. The Sister fork ratio is calculated as the ratio of the length of a longer IdU track over a shorter IdU track in each bidirectional fork. A ratio of ~1 indicates fork symmetry, while >1 indicates fork asymmetry. (Middle) Representative DNA fibre images depicting symmetric and asymmetric replication tracts on sister forks. (Bottom) Quantification of fork asymmetry in control and RECQL5-depleted U2OS cells. A total of ≥33 DNA fibers emanating from a single origin were analysed across three biological replicates. Data is represented as mean ±SEM. Mann-Whitney t test, *p<0.05; **p<0.001; ***p<0.0001; ****p<0.0001; ns, non-significant.

(D) (Top) Schematic of labeling protocol for scoring fork restart events by DNA fiber assay. (Middle) Representative DNA fibers show a stalled fork (red only tract), restarted fork (red followed by a green tract). (Bottom) Bar plot showing the percentage of stalled and restarted forks in control and RECQL5-depleted U2OS cells. A total of ≥300 DNA fibers were analysed across three biological replicates. Data is represented as mean ±SEM. One-way ANOVA test, *p<0.05; **p<0.001; ***p<0.0001; ****p<0.0001; ns, non-significant.

(E) (Left) Representative DNA fibers showing IdU tract lengths in the restarted forks. (Right) Quantification of IdU track lengths of the restarted forks in control and RECQL5-depleted U2OS cells. A total of ≥300 DNA fibers were analysed across three biological replicates. Data is represented as mean ±SEM. Mann-Whitney t test, *p<0.05; **p<0.001; ***p<0.0001; ****p<0.0001; ns, non-significant.

Supplementary Figure 2

(A) Representative images of chromatin-bound γH2AX and 53BP1 foci in control and RECQL5-depleted U2OS cells. Cells were treated with 1mM HU for 4 h and recovered in fresh media for indicated times prior to pre-extraction and fixation, followed by immunofluorescence staining. Nuclear boundaries are marked with white-dashed lines. Scale bar, 5µm.

(B) Quantification of chromatin-bound γH2AX foci/nucleus for conditions as shown in (A). A total of ≥225 nuclei were analysed across three biological replicates. Data is represented as mean ±SEM. Mann-Whitney t test, *p<0.05; **p<0.001; ***p<0.0001; ****p<0.0001; ns, non-significant.

(C) Quantification of chromatin-bound 53BP1foci/nucleus for conditions as shown in (A). A total of ≥225 nuclei were analysed across three biological replicates. Data is represented as mean ±SEM. Mann-Whitney t test, *p<0.05; **p<0.001; ***p<0.0001; ****p<0.0001; ns, non-significant.

(D) (Left) Representative images of neutral comet tails in control and RECQL5-depleted U2OS cells in untreated or treated with 1mM HU for 4 h. (Right) Quantification of comet tail moment in indicated U2OS cells. A total of ≥150 comet tails were analysed across three biological replicates. Data is plotted as a box plot. The centre line indicates the median value; the boxes indicate the 25th and 75th percentiles, while the whiskers indicate the 10th and 90th percentiles. Points below and above the whiskers are plotted as individual points. Mann-Whitney t test, *p<0.05; **p<0.001; ***p<0.0001; ****p<0.0001; ns, non-significant.

(E) Survival analysis in control and RECQL5-depleted U2OS cells treated with the indicated dose of HU for 24 h. Cell survival is expressed as a percentage relative to untreated control. Data represented as ± SD from three independent experiments.

(F) Survival analysis in control and RECQL5-depleted U2OS cells treated continuously with the indicated dose of Aphidicolin. Cell survival is expressed as a percentage relative to untreated control. Data represented as ± SD from three independent experiments.

(G) Representative immunoblot showing expression of shRNA resistant FLAG-tagged RECQL5 in RECQL5-depleted U2OS cells transfected with empty vector, WT, PIPLA and PIPA mutants. β ACTIN serves as a loading control.

Supplementary Figure 3

(A) Representative immunoblot showing depletion of RECQL5 and RAD51 in U2OS cells after transfection with indicated shRNAs. MCM3 serves as a loading control.

(B) Representative immunoblot showing expression of shRNA-resistant FLAG-tagged RAD51 in U2OS cells transfected with empty vector, WT, T131P, and IIA RAD51 mutants in RAD51 alone and RECQL5-RAD51 co-depleted cells. GAPDH serves as a loading control.

(C) Representative immunoblot showing depletion of RECQL5 and HLTF in U2OS cells after transfection with indicated shRNAs. * represents a non-specific band. LAMIN serves as a loading control.

(D) Representative immunoblot showing depletion of ZRANB3 in indicated U2OS cells after transfection with indicated shRNAs. LAMIN serves as a loading control.

(E) Representative immunoblot showing depletion of SMARCAL1 in indicated U2OS cells after transfection with indicated shRNAs. LAMIN serves as a loading control.

(F) Representative immunoblot showing depletion of FBH1 in indicated U2OS cells after transfection with indicated shRNAs. LAMIN serves as a loading control.

(G) (Left)(Top) Schematic of labeling scheme used for DNA fiber assay. (Bottom) Representative DNA fiber images to show replication rates in control, RECQL5 alone and RECQL5 co-depleted with fork reversal factor HLTF in hTERT-RPE1 cells. (Right) Quantification of IdU track lengths for indicated conditions. A total of ≥225 DNA fibers were analysed across two biological

replicates. Data is represented as mean ±SEM. Mann-Whitney t test, *p<0.05; **p<0.001; ***p<0.0001; ****p<0.0001; ns, non-significant.

(H) Quantification of comet tail moment in control, RECQL5 alone and RECQL5 co-depleted with fork reversal factors in U2OS cells as indicated. A total of ≥150 comet tails were analysed across three biological replicates. Data is plotted as a box plot. The centre line indicates the median value; the boxes indicate the 25th and 75th percentiles, while the whiskers indicate the 10th and 90th percentiles. Points below and above the whiskers are plotted as individual points. Mann-Whitney t test, *p<0.05; **p<0.001; ***p<0.0001; ****p<0.0001; ns, non-significant.

Supplementary Figure 4

(A) Representative immunoblot showing depletion of RECQL5 and RAD51C in U2OS cells after transfection with indicated shRNAs. MCM3 serves as a loading control.

(B) Representative immunoblot showing depletion of RECQL5 and XRCC2 in U2OS cells after transfection with indicated shRNAs. MCM3 serves as a loading control.

(C) Representative immunoblot showing depletion of RECQL5 and XRCC3 in U2OS cells after transfection with indicated shRNAs. MCM3 serves as a loading control.

(D) (Left) (Top) Schematic of labeling scheme used for DNA fiber assay. (Bottom) Representative DNA fiber images to show replication rates in control, RECQL5 alone and RECQL5 co-depleted with indicated HR factors in hTERT-RPE1 cells. (Right) Quantification of IdU track lengths for indicated conditions. A total of ≥225 DNA fibers were analysed across two biological replicates. Data is represented as mean ±SEM. Mann-Whitney t test, *p<0.05; **p<0.001; ***p<0.0001; ****p<0.0001; ns, non-significant.

(E) (Left) (Top) Schematic of labeling protocol for scoring fork restart events by DNA fiber assay. (Bottom) Representative DNA fibers showing IdU tract lengths in the restarted forks. (Right) Quantification of IdU track lengths of the restarted forks in indicated U2OS cells. A total of ≥300 DNA fibers were analysed across three biological replicates. Data is represented as mean ±SEM. Mann-Whitney t test, *p<0.05; **p<0.001; ***p<0.0001; ****p<0.0001; ns, non-significant.

(F) A schematic of SCR reporter depicting the mechanism of HR upon DSB induced by I-SceI expression.

(G) Quantification of I-SceI-induced HR frequencies in control, RECQL5 alone and RECQL5 co-depleted with indicated HR factors in U2OS SCR24 cells. Data is represented as mean ±SEM from three independent experiments. One-way ANOVA test, *p<0.05; **p<0.001; ***p<0.0001; ****p<0.0001; ns, non-significant.

Supplementary Figure 5

(A) Representative immunoblot showing expression of shRNA-resistant hemagglutinin (HA) tagged XRCC3 in RECQL5-XRCC3 co-depleted U2OS cells transfected with empty vector, WT and S225A mutant of XRCC3. αTUBULIN serves as a loading control.

(B) (Top) Schematic of labeling scheme used for DNA fiber assay. (Middle) Representative DNA fiber images showing ongoing replication in U2OS cells expressing shRNA-resistant XRCC3

variants in RECQL5-XRCC3 co-depleted cells. (Bottom) Quantification of IdU track lengths as replication rate (kb/min) for indicated conditions. A total of ≥300 DNA fibers were analysed across three biological replicates. Data is represented as mean ±SEM. Mann-Whitney t test, *p<0.05; **p<0.001; ***p<0.0001; ****p<0.0001; ns, non-significant.

(C) Representative immunoblot showing expression of shRNA-resistant HA-tagged XRCC2 in RECQL5-XRCC2 co-depleted U2OS cells transfected with empty vector, WT and S247A mutant of XRCC2. αTUBULIN serves as a loading control.

(D) (Top) Schematic of labeling scheme used for DNA fiber assay. (Middle) Representative DNA fiber images showing ongoing replication in U2OS cells expressing shRNA-resistant XRCC2 variants in RECQL5-XRCC2 co-depleted cells. (Bottom) Quantification of IdU track lengths as replication rate (kb/min) for indicated conditions. A total of ≥300 DNA fibers were analysed across three biological replicates. Data is represented as mean ±SEM. Mann-Whitney t test, *p<0.05; **p<0.001; ***p<0.0001; ****p<0.0001; ns, non-significant.

(E) Representative images of chromatin-bound XRCC3-SIRF signals at progressing, stalled, and recovery conditions in control and RECQL5 depleted cells. HU-treated cells were washed thrice with 1X PBS and recovered in fresh media for 3 h and 6 h to assess XRCC3 persistence at stalled fork sites. Scale bar, 5µm.

(F) Quantification of chromatin-bound XRCC3-SIRF signals as shown in (E). A total of ≥300 nuclei were analysed across three biological replicates. Data is represented as mean ±SEM. Mann-Whitney t test, *p<0.05; **p<0.001; ***p<0.0001; ****p<0.0001; ns, non-significant.

Supplementary Figure 6

(A) Representative images of chromatin-bound γH2AX foci in indicated U2OS cells. Nuclear boundaries are marked with white-dashed lines. Scale bar, 5µm.

(B) Quantification of chromatin-bound γH2AX foci/nucleus for conditions as shown in (A). A total of ≥225 nuclei were analysed across three biological replicates. Data is represented as mean ±SEM. Mann-Whitney t test, *p<0.05; **p<0.001; ***p<0.0001; ****p<0.0001; ns, non-significant.

(C) (Left) Representative image showing micronucleation in U2OS cell. Scale bar, 5µm. (Right) Bar plot showing the percentage of micro-nucleated cells in the indicated U2OS cells. A total of ≥225 nuclei were analysed across three biological replicates. Data is represented as mean ±SEM. Mann-Whitney t test, *p<0.05; **p<0.001; ***p<0.0001; ****p<0.0001; ns, non-significant.

(D) Representative images of a metaphase spread in indicated U2OS cells. Red arrows indicate chromosomal aberrations (breaks, gaps and radials). Cells were treated with 2mM HU for 4 h and recovered in fresh media for 24 h.

(E) Quantification of chromosomal aberrations (breaks, gaps and radials) in indicated cells shown in (D). A total of ≥75 spreads were analysed across three biological replicates. Data is represented as mean ±SD. Mann-Whitney t test, *p<0.05; **p<0.001; ***p<0.0001; ****p<0.0001; ns, non-significant.

(F) Quantification of BrdU foci/nucleus in indicated U2OS cells. Cells were labeled with 25 µM BrdU for 24h, washed thrice with 1X PBS and treated with 2 mM HU for 4 h. Samples were then processed for immunofluorescence analysis in non-denaturing conditions. A total of ≥225 nuclei were analysed across three biological replicates. Data is represented as mean ±SEM. Mann-Whitney t test, *p<0.05; **p<0.001; ***p<0.0001; ****p<0.0001; ns, non-significant.

(G) Bar plot showing percentage survival of indicated U2OS cells upon exposure to 2 mM HU for 4 h. Cell survival is expressed as a percentage relative to untreated control. Data represented as ± SD from three independent experiments. Unpaired t test, *p<0.05; **p<0.001; ***p<0.0001; ****p<0.0001; ns, non-significant.

Supplementary Figure 7

(A) Representative immunoblot showing expression of shRNA-resistant FLAG-tagged RECQL5 in RECQL5-depleted U2OS cells transfected with empty vector, WT, K58R and F666A mutants. LAMIN serves as a loading control.
(B) (Top) Schematic of labeling protocol used for scoring fork restart events by DNA fiber assay. (Bottom) Bar plot showing the percentage of stalled forks, restarted forks and new origin firing events in indicated U2OS cells. A total of ≥300 DNA fibers were analysed across three biological replicates. Data is represented as mean ±SEM. One-way ANOVA test, *p<0.05; **p<0.001; ***p<0.0001; ****p<0.0001; ns, non-significant.
(C) (Top) Representative DNA fibers showing IdU tract lengths in the restarted forks in the indicated cells. (Bottom) Quantification of IdU track lengths of the restarted forks as indicated on the left. A total of ≥300 DNA fibers were analysed across three biological replicates. Data is represented as mean ±SEM. Mann-Whitney t test, *p<0.05; **p<0.001; ***p<0.0001; ****p<0.0001; ns, non-significant.
(D) Representative immunoblot showing expression of shRNA-resistant FLAG-tagged RECQL5 in RECQL5-depleted U2OS cells transfected with empty vector, WT and S727A mutant. β ACTIN serves as a loading control.
(E) (Left)(Top) Schematic of labeling scheme used for DNA fiber assay. (Left)(Bottom) Representative DNA fiber images showing replication rate in indicated U2OS cells. (Right) Quantification of IdU track lengths as replication rate (kb/min) for indicated conditions. A total of ≥300 DNA fibers were analysed across three biological replicates. Data is represented as mean ±SEM. Mann-Whitney t test, *p<0.05; **p<0.001; ***p<0.0001; ****p<0.0001; ns, non-significant.
(F) Representative immunoblot showing depletion of RTEL1 in indicated U2OS cells after transfection with indicated shRNAs. αTUBULIN serves as a loading control.
(G) Representative images of chromatin-bound RAD51-SIRF signals at progressing, stalled, and recovering forks in indicated cells. HU-treated cells were washed thrice with 1X PBS and recovered in fresh media for 4 h to assess RAD51 persisting at stalled fork sites. Scale bar, 5µm.

Supplementary Figure 8

(A) Representative immunoblot showing expression of shRNA-resistant FLAG-tagged RECQL5 in RECQL5-depleted U2OS cells transfected with empty vector, WT, K598E, R943A and K598E+P908X mutants. LAMIN serves as a loading control.

(B) Representative immunoblot showing expression of shRNA-resistant FLAG-tagged RECQL5 in RECQL5-depleted U2OS cells transfected with empty vector, WT, K598E, R943A, K598E+P908X, F666A, K598E+F666A+P908X mutants. β ACTIN serves as a loading control.

Supplementary Table 1: Sequences of shRNAs used in this study

Gene	shRNA sequence (5'-3')	References
RECQL5 shRNA#1 (Gene specific)	GGAGAGTGCGACCATGGC	1
RECQL5 shRNA#2 (Gene specific)	CAGGTTTGTCGCCCATTGGAA	1
RTEL1	GAGAAGCCCTGAGCTACCTTGGGGT	2
RAD51C	CACCTTCTGTTCAGCACTAGA	3
XRCC2	TTGCAACGACACAAACTATAA	4
XRCC3	GAATTATTGCTGCAATTAA	3
RAD51	GAAGAAATTGGAAGAAGCT	3
SMARCAL1	GCTTTGACCTTCTTAGCAAT	5
ZRANB3	TGGTGTGTGTCAGCTCTGT	6
HLTF	GGAATATAATGTTAACGAT	7
FBH1	AAACAAAACCTCGTCATTA	8

Supplementary Table 1 references:

1. Urban, Vaclav et al. "RECQ5 helicase promotes resolution of conflicts between replication and transcription in human cells." The Journal of cell biology vol. 214,4 (2016): 401-15. doi:10.1083/jcb.201507099
2. Wu, Wei et al. "RTEL1 suppresses G-quadruplex-associated R-loops at difficult-to-replicate loci in the human genome." Nature structural & molecular biology vol. 27,5 (2020): 424-437. doi:10.1038/s41594-020-0408-6
3. Somyajit, Kumar et al. "ATM- and ATR-mediated phosphorylation of XRCC3 regulates DNA double-strand break-induced checkpoint activation and repair." Molecular and cellular biology vol. 33,9 (2013): 1830-44. doi:10.1128/MCB.01521-12
4. Saxena, Sneha et al. "XRCC2 Regulates Replication Fork Progression during dNTP Alterations." Cell reports vol. 25,12 (2018): 3273-3282.e6. doi:10.1016/j.celrep.2018.11.085
5. Postow, Lisa et al. "Identification of SMARCAL1 as a component of the DNA damage response." The Journal of biological chemistry vol. 284,51 (2009): 35951-61. doi:10.1074/jbc.M109.048330
6. Ciccia, Alberto et al. "Polyubiquitinated PCNA recruits the ZRANB3 translocase to maintain genomic integrity after replication stress." Molecular cell vol. 47,3 (2012): 396-409. doi:10.1016/j.molcel.2012.05.024
7. Kang, Zhihua et al. "BRCA2 associates with MCM10 to suppress PRIMPOL-mediated repriming and single-stranded gap formation after DNA damage." Nature communications vol. 12,1 5966. 13 Oct. 2021, doi:10.1038/s41467-021-26227-6
8. Fugger, Kasper et al. "FBH1 Catalyzes Regression of Stalled Replication Forks." Cell reports vol. 10,10 (2015): 1749-1757. doi:10.1016/j.celrep.2015.02.028

Supplementary Table 2: Primer sequences used to generate shRNA#1 resistant-WT and mutant RECQL5 plasmid constructs in the study. The nucleotide changes required to introduce necessary mutations are bolded and underlined.

Primer name	Sequence (5’-3’)
Q5 Kpn1 FP	TTTTGGTACCATGAGCAGCCACCATACCACCTTTCC
Q5 EcoR1 FLAG RP	TTTTGAATTCTTACTTATCGTCGTCATCCTTGTAATCTCTCTGGGGGCCACACAGGCCATGC
Q5 sh1 Res FP	CTTTTAAGACGCCTTTACA**A**GA**A**AG**C**GC**C**AC**A**ATGGCTGTAGTAAAAGGTAAC
Q5 sh1 Res RP	GTTACCTTTTACTACAGCCAT**T**GT**G**GC**G**CT**T**TC**T**TGTAAAGGCGTCTTAAAAG
PIPL3A FP	GCGGCCCACAAGGATTCTCAGAGC**GC**CGCCCGC**GCCGC**CTGCCGAAGGGTGGAAAGCCCAGCTC
PIPL3A RP	GAGCTGGGCTTTCCACCCTTCGGCAG**GCGGC**GCGGGCG**GC**GCTCTGAGAATCCTTGTGGGCCGC
Q5 R943A FP	GTTGTTTAAAGGCTTTGCC**GCT**CACCTCTCACACTTGC
Q5 R943A RP	GCAAGTGTGAGAGGTG**AGC**GGCAAAGCCTTTAAACAAC
Q5 PIP 4A FP	GAGCGTGAAAGAAGAGGCC**GCT**AACCTC**GCC**AGGCAC**GCTGCT**CATGGCCGGGCCCG
Q5 PIP4A RP	CGGGCCCGGCCATG**AGCAGC**GTGCCT**GGC**GAGGTT**AGC**GGCCTCTTCTTTCACGCTC
Q5 K598E FP	AAGGTGGCCAACCTCTAC**GAG**GCCAGCGTGCTGAAGAAGGTGGCCGAT
Q5 K598E RP	ATCGGCCACCTTCTTCAGCACGCTGGC**CTC**GTAGAGGTTGGCCACCTT
Q5 K58R FP	CATGCCCACAGGGGCAGGA**AGA**TCCCTATGCTATCAGCTC
Q5 K58R RP	GAGCTGATAGCATAGGGA**TCT**TCCTGCCCCTGTGGGCATG
Q5 F666A FP	CCCAAAGGCTCCTGCCCG**GCT**CAGACGGCCACGGAACTG
Q5 F666A RP	CAGTTCCGTGGCCGTCTG**AGC**CGGGCAGGAGCCTTTGGG
Q5 P908X FLAG RP	GCGCGAATTCTTACTTATCGTCGTCATCCTTGTAATCAGGAGCGGAGAGCTGGAAGGGGTCTTG

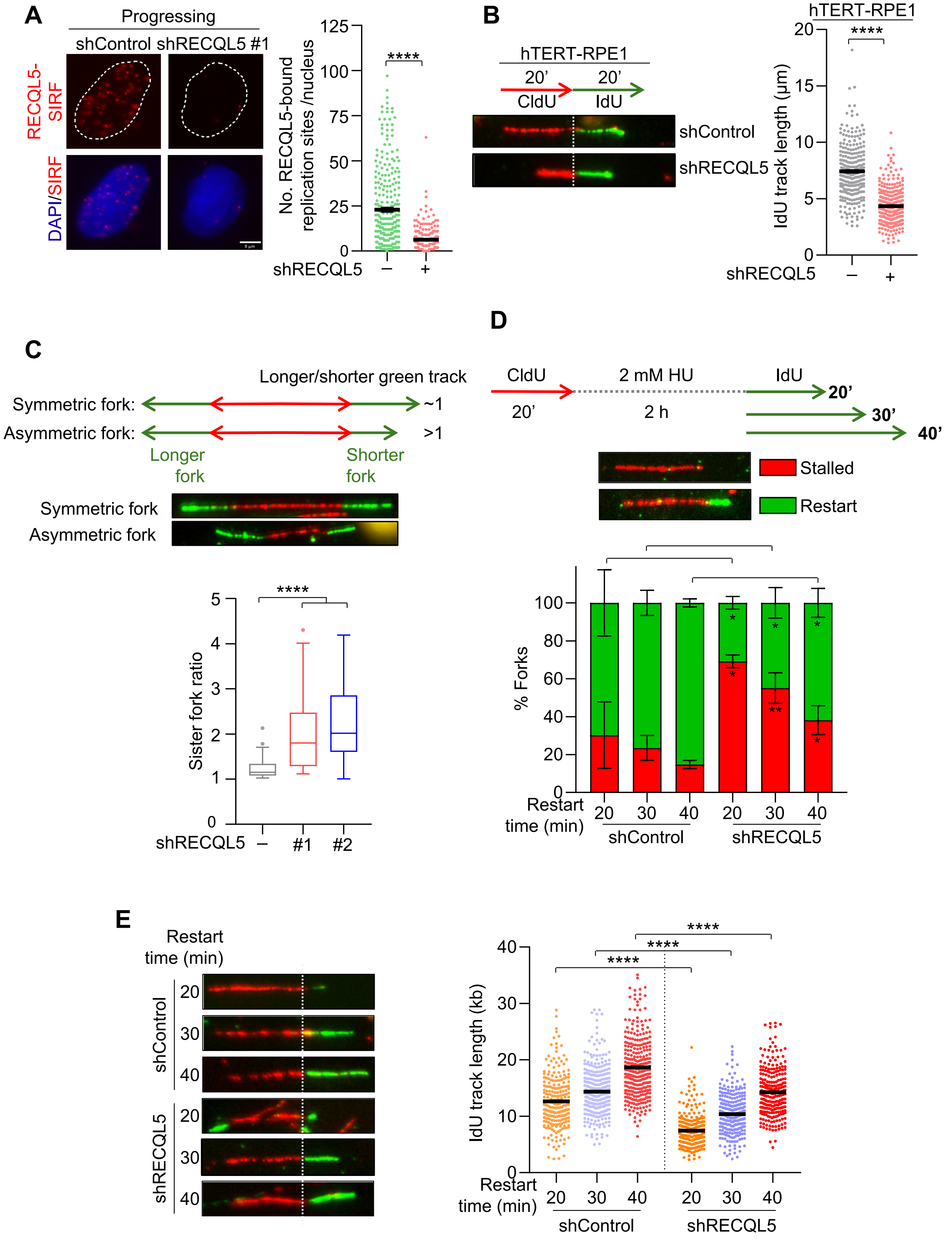

Supplementary figure 1

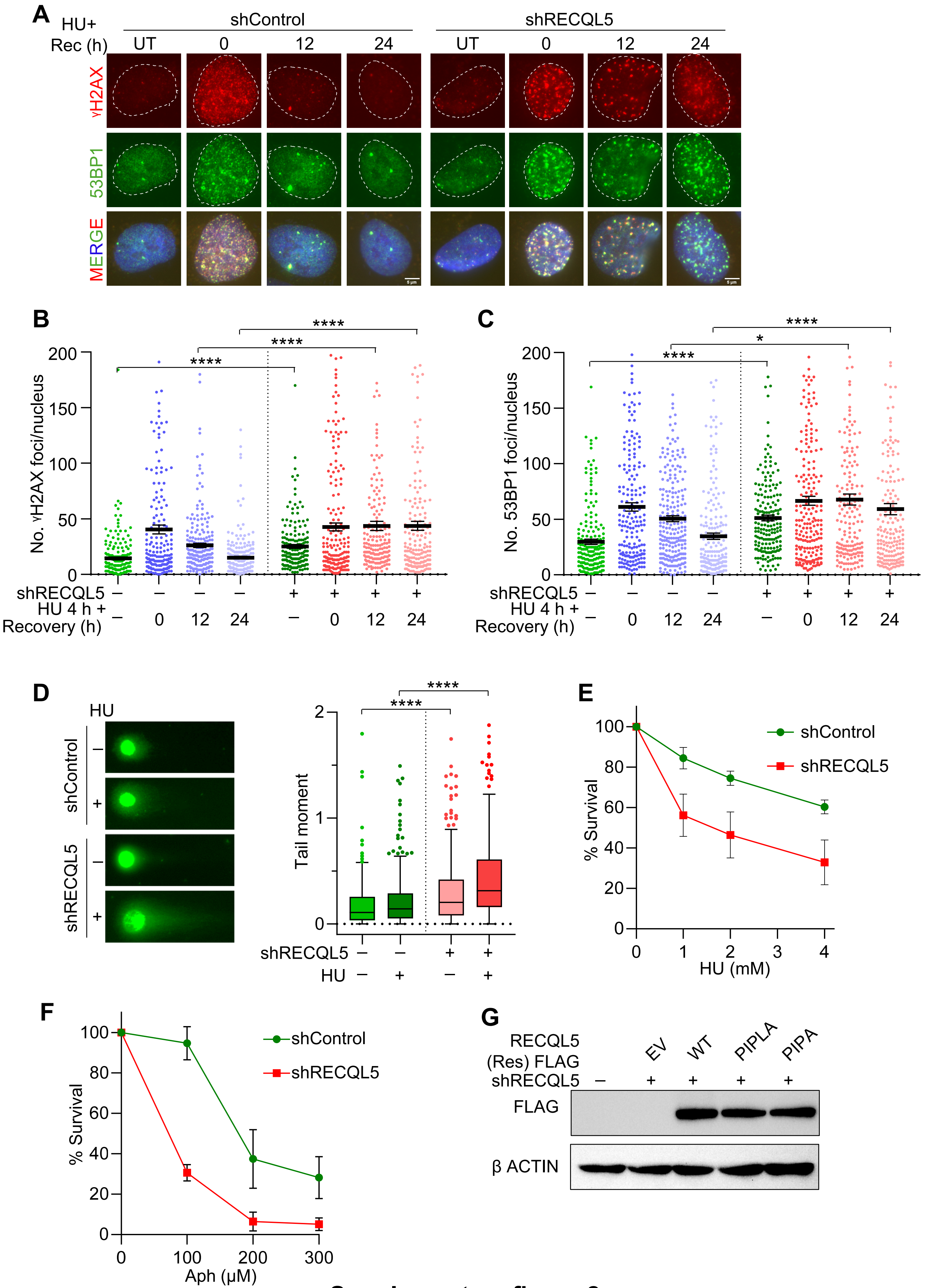

Supplementary figure 2

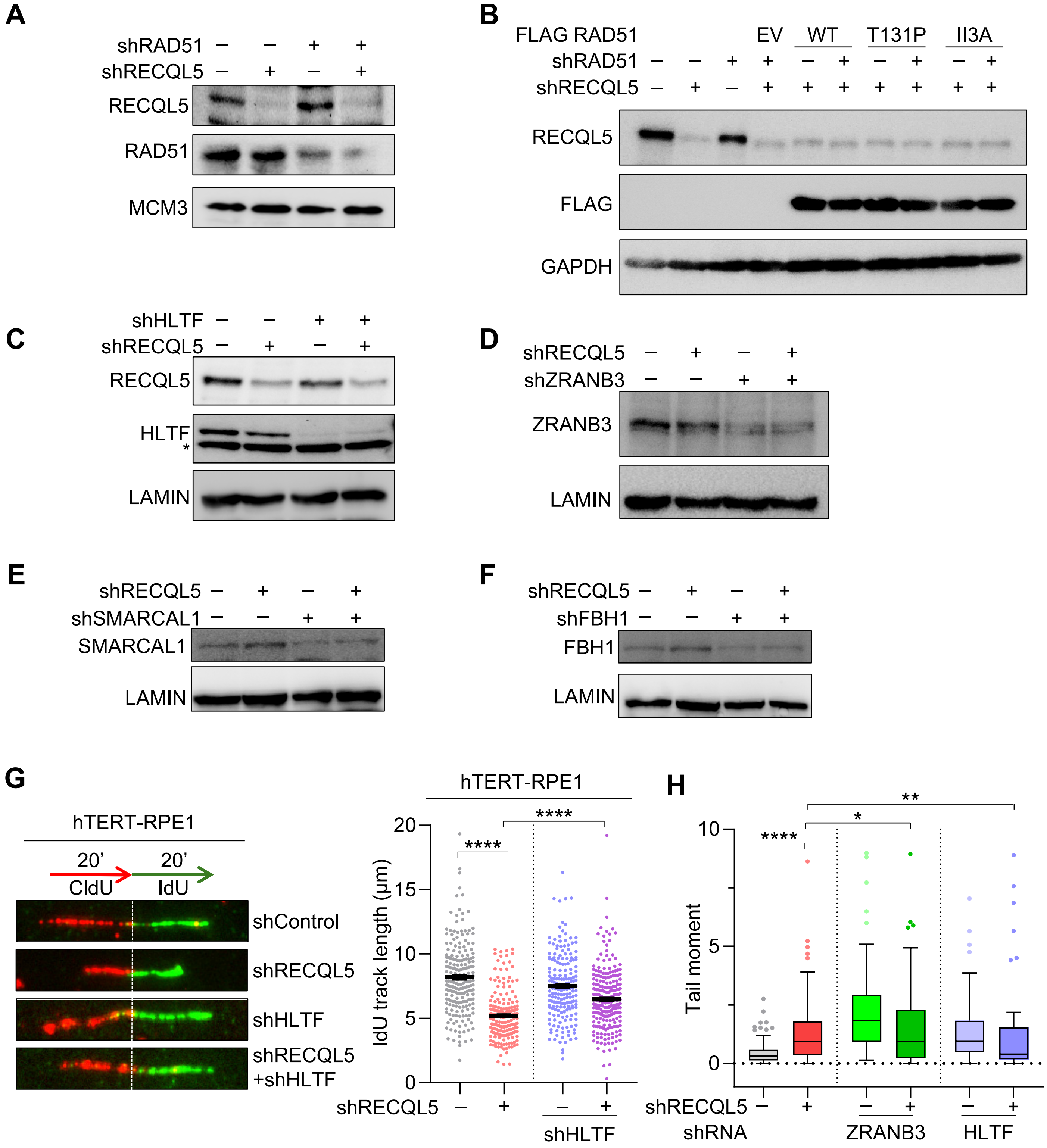

Supplementary figure 3

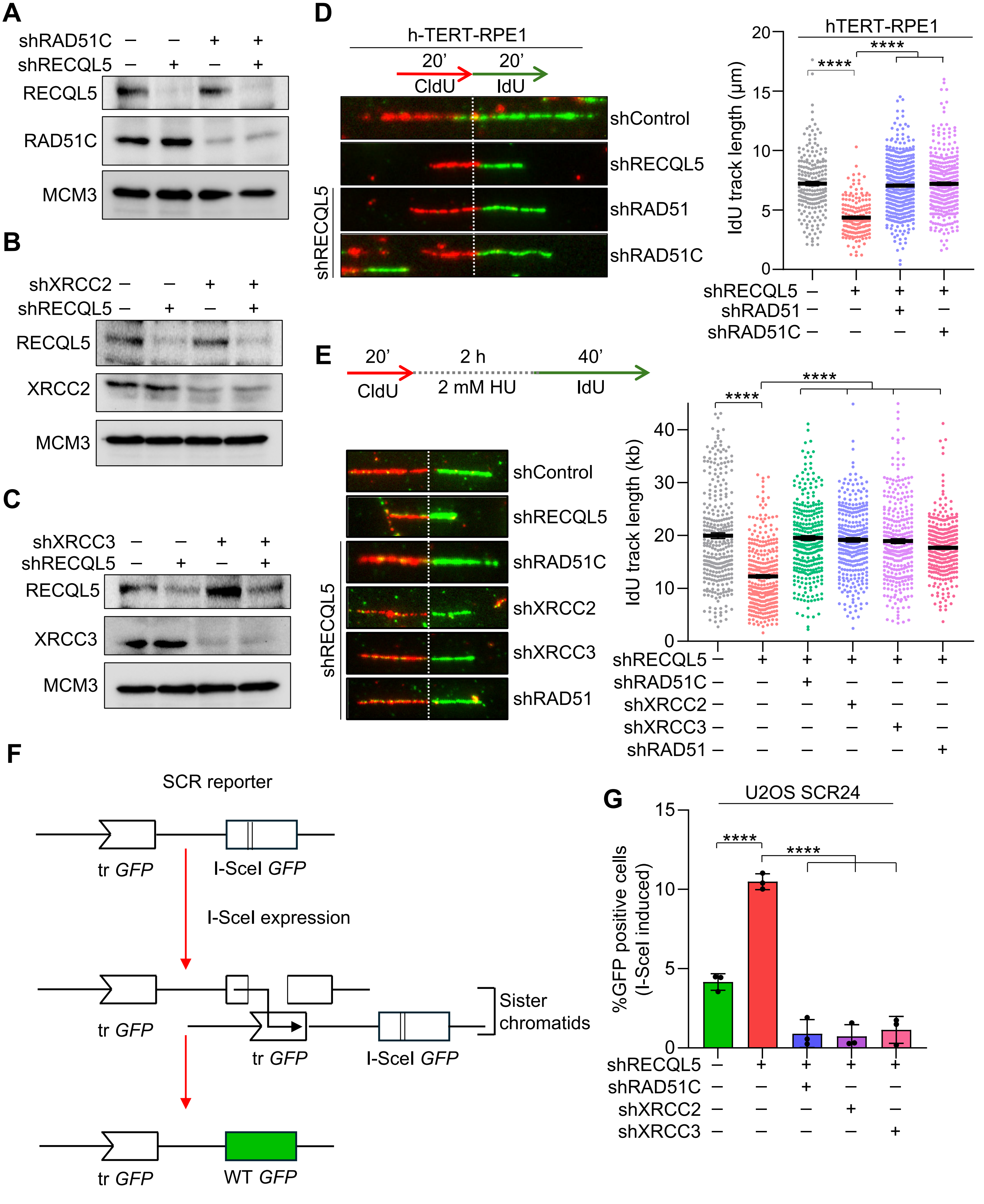

Supplementary figure 4

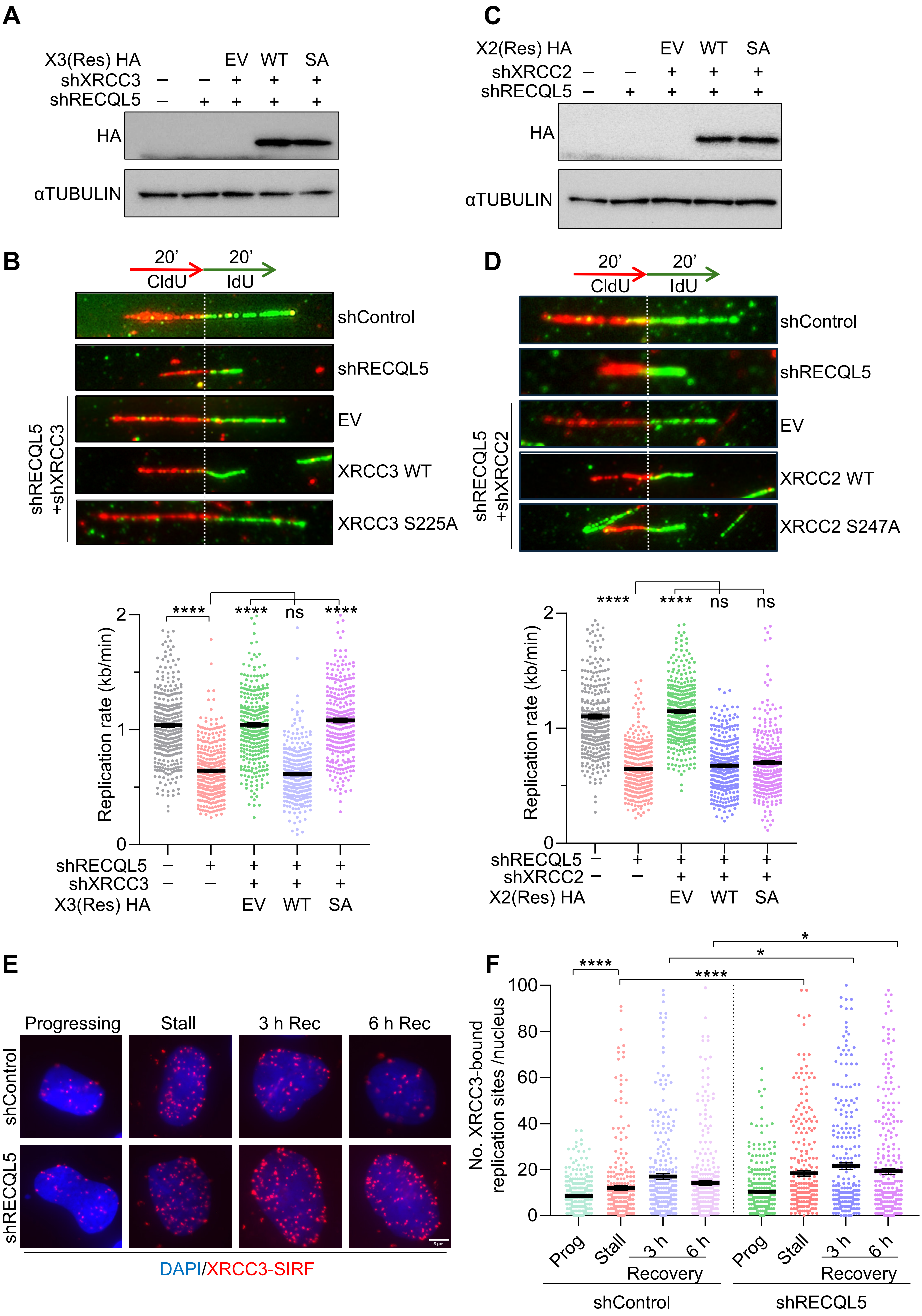

Supplementary figure 5

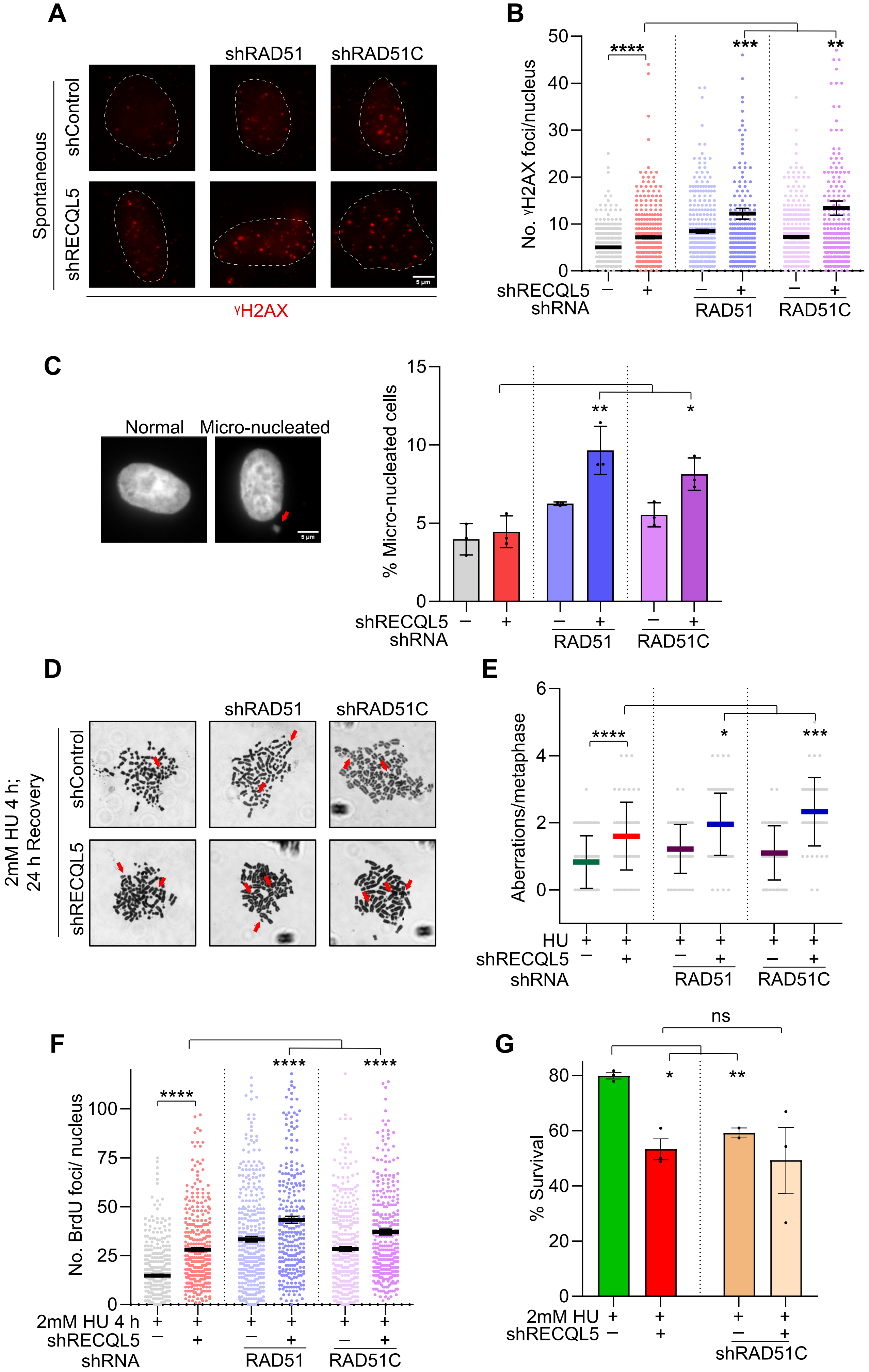

Supplementary figure 6

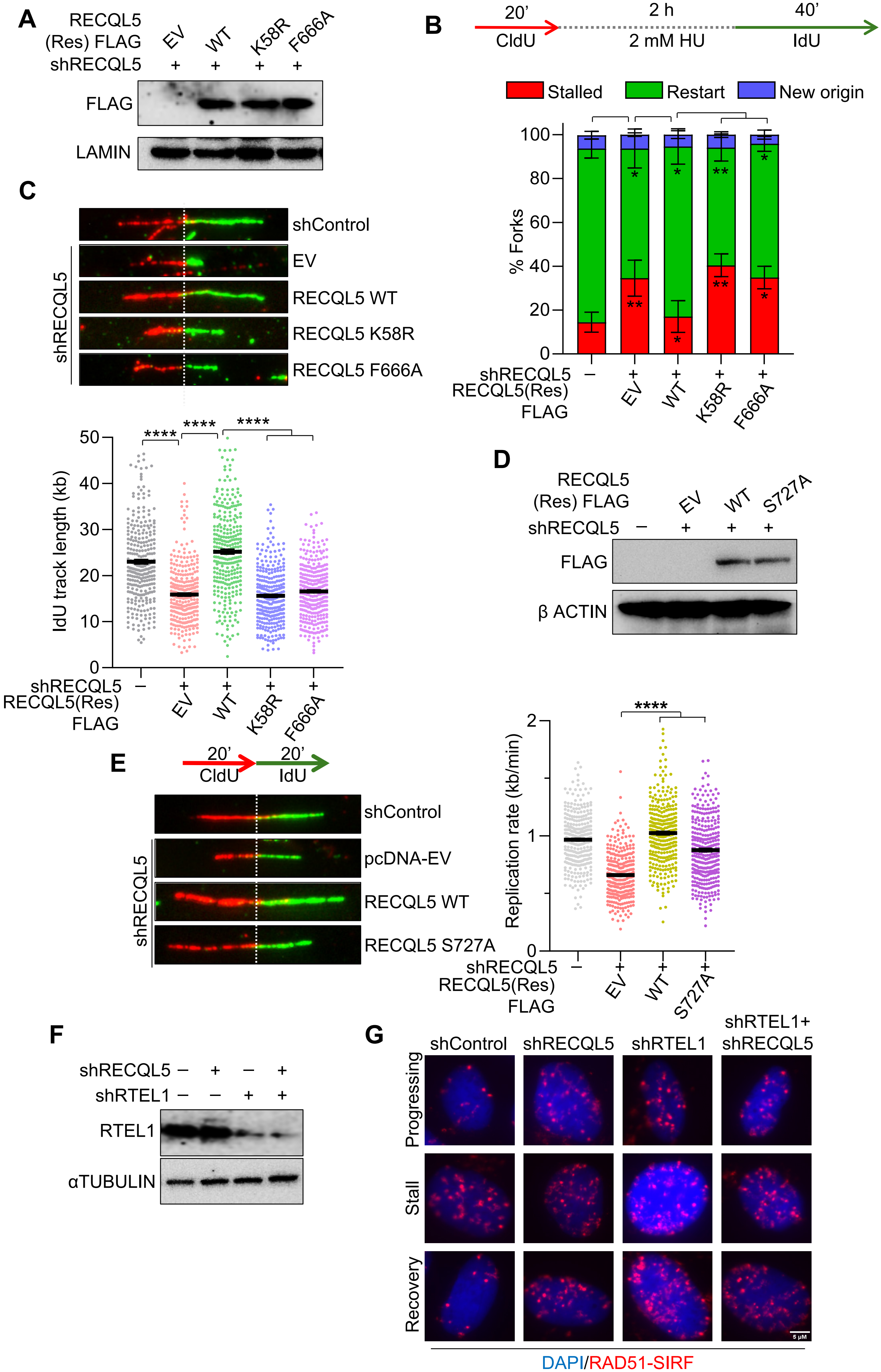

Supplementary figure 7

A

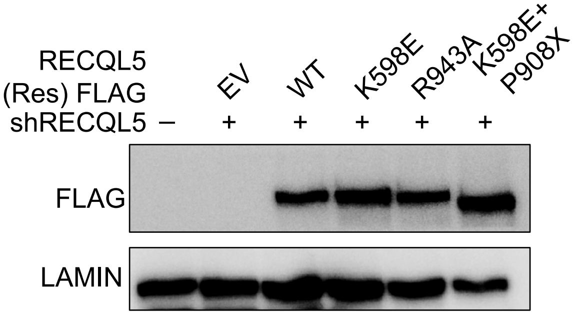

B

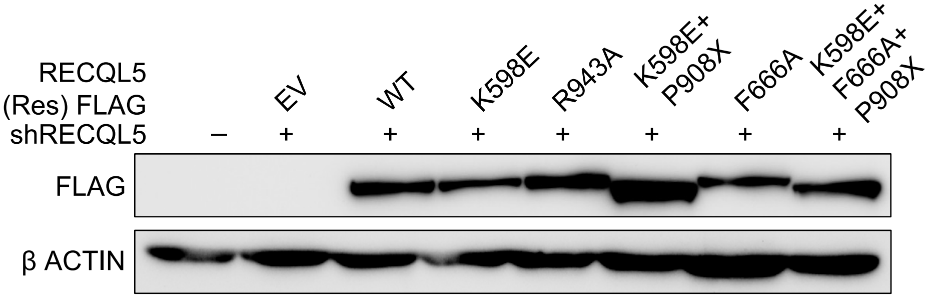

Supplementary figure 8
